# Regeneration of Ni–Zr Methane Dry Reforming Catalysts in CO_2_: Reduction of Coking and Ni Redispersion

**DOI:** 10.1021/acscatal.4c06230

**Published:** 2025-02-10

**Authors:** Mahdi Hosseinpour, Toni Moser, Bernhard Klötzer, Simon Penner

**Affiliations:** Institute of Physical Chemistry, University of Innsbruck, Innrain 52c, Innsbruck 6020, Austria

**Keywords:** catalyst regeneration, intermetallic precursor, CO_2_, sintering, carbon deposition, redispersion, reverse Boudouard reaction

## Abstract

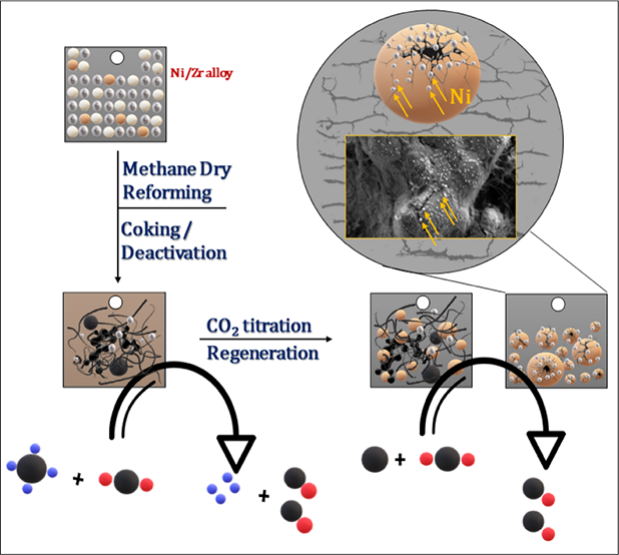

Bimetallic Ni–Zr layers on top of a Zr metal substrate are oxidatively transformed into an active Ni metal–ZrO_2_ interfacial state under methane dry reforming (DRM) conditions, which exhibits progressive coking with increasing DRM cycle number and time. Complementing established anticoking catalyst- and process-design strategies, the efficiency, as well as the structural and surface-chemical consequences of the intermediate regeneration of the active state using pure CO_2_, were studied. By combining repeated catalytic DRM testing, X-ray photoelectron spectroscopy, and chemically resolved scanning electron microscopy, we show that catalyst regeneration using the inverse Boudouard reaction not only depletes the main part of the deposited coke efficiently but also leads to an improved and particularly active catalyst state exhibiting redispersion of Ni toward small particles and optimized Ni/ZrO_2_ interfacial dimensions.

## 1 Introduction

Dry reforming of methane (DRM), CH_4_ + CO_2_ → 2H_2_ + 2CO, is a catalytic process that converts methane (CH_4_) and carbon dioxide (CO_2_) into synthesis gas (syngas), a mixture of hydrogen (H_2_) and carbon monoxide (CO). It provides a viable way for converting harmful greenhouse gases into syngas with a maximum H_2_/CO ratio of unity.^1^ Despite its benefits, DRM faces notable challenges: catalyst deactivation due to carbon deposition (i.e., coking), sintering of active metal particles, and insufficient H_2_-selectivity are major obstacles that need to be addressed to make DRM a practical industrial process.^2,3^

Both noble and base metals can be used as promising active components in DRM. However, the large number of catalysts required for industrial methane dry reforming makes only base-metal catalysts, such as nickel (Ni) and cobalt (Co), economically viable.^4^ Ni-based catalysts are especially known for their high activity and ability to facilitate the dissociation of methane and carbon dioxide, making them highly effective for syngas production.^5^ Despite their advantages, pure Ni catalysts are susceptible to significant deactivation under DRM conditions due to pronounced coking and sintering of active metal particles.^6^ To solve these problems, various strategies were investigated, including the addition of supports like alumina,^7^ silica,^8^ and zirconia^9^ to increase the thermal stability and dispersion of the Ni particles. Strongly connected to the tailoring of the dispersion of metals, including Ni, is the use of catalytic precursor materials, whose decomposition can be steered to engineer the morphology, anchoring, or dispersion of the metal particles. A material class, where this strategy is particularly worthwhile, is metallic glasses,^10,11^ alloys,^12^ and intermetallic compounds.^13−15^ Especially the latter, thanks to their highly uniform atomic arrangement and electronic structure, are a current hot topic in heterogeneous catalysis.^13−15^ In particular, both alloys and intermetallic compounds have been widely used as catalyst precursor materials to design the metal-oxide interface resulting from the decomposition of the precursor structures. This strategy was shown, e.g., for methanol steam or methane dry reforming to lead to catalysts with enhanced selectivity, activity and stability.^16^ For DRM applications, we have revealed this specifically for Ni- and Pd-based Zr alloys and intermetallic compounds. Both decompose into the respective Ni-ZrO_2_ and Pd-ZrO_2_ systems upon activation, as proven for both industrially relevant powder materials and thin film samples.^1,9,17−19^ The latter, which forms the core of the present contribution, in principle, allows for the coating of metal-based monolithic catalysts and the reactive formation of active alloys or intermetallic compound coatings. This is very important, as monolithic catalyst activation strategies offer efficient gas–solid interactions and reduced pressure drop during reactions, making them ideal for large-scale industrial applications.^20^

These innovative approaches enable the design of high-performance catalysts that address common challenges, especially in catalyst deactivation, such as sintering and coke formation, ultimately extending catalyst lifetime and efficiency in various chemical processes.^21^ Regeneration of catalysts deactivated by coke or carbon, which is the most important issue in DRM, can be done by gasification with oxygen, water, hydrogen, or carbon dioxide. Although of course dependent on the specific conditions, a generally valid trend in reaction rates of carbon gasification is O_2_ > H_2_O > CO_2_ > H_2_. The efficiency of coke and carbon removal is also strongly dependent on the type of carbonaceous species: more graphitic or less-reactive carbon species require very high temperatures (>700 °C) in either H_2_O or H_2_, again raising the issue of increased metal particle sintering.^21^ With respect to process efficiency, oxygen is clearly the most potent, but for DRM applications is less favorable on Ni-based catalysts due to the possibility of oxidation of the Ni particles (in due course deactivating the catalyst) and, generally, gas switching from DRM to oxygen. Herein, we report a detailed account of using carbon dioxide as a carbon gasification agent, which suppresses Ni oxidation and promotes process efficiency likewise.

Additionally, promoters such as cerium and lanthanum have been investigated to improve resistance to coking and enhance the overall catalytic activity.^22,23^ Intermetallic Ni–Zr catalysts have also shown promise in improving the resistance to coking and maintaining structural integrity.^24^ To enhance further the coke resistance several approaches were followed. Odedairo et al. reported that plasma treatment of Ni/CeO_2_ led to higher activity in methane dry reforming by increasing the metal–support interface and generating a clean metal surface.^25^ Németh et al. reported that incorporating sodium as a promoter in a Ni–Zr catalyst can significantly enhance its catalytic activity by resistance to coking.^26^ Conversely, Wei et al. indicated that the activity was only related to the type of the active metal and its particle size, which made the reaction conditions the most important factor for the stability of the catalyst.^27^ Regeneration of coked catalysts, especially in nickel-based systems, is crucial for maintaining their long-term efficiency in processes such as dry reforming of methane.^28^ Coking on the catalyst surface deactivates Ni-based catalysts by blocking active sites and promoting particle sintering.^29^ One of the key factors influencing the coking propensity of Ni catalysts is particle size:^30^ smaller Ni particles generally exhibit greater resistance to coke formation due to their higher surface energy and ability to disperse carbon atoms.^31^ Larger Ni particles are more prone to carbon deposition, leading to quicker deactivation.^32^ Regeneration techniques, such as oxidative treatments or CO_2_ titration, aim to remove accumulated carbon while preserving the structural integrity of the catalyst.^1^ These approaches, drawn from comprehensive DRM research, emphasize the importance of managing particle size and adopting efficient regeneration methods to enhance the durability of Ni-based catalysts.

Herein, we focus on bimetallic Ni–Zr catalysts, which give rise to very active DRM catalysts when activated directly in the DRM mixture through the formation of a Ni-ZrO_2_ interface. In due course, we reveal how CO_2_ treatment is a very promising regeneration strategy for deactivated Ni-based catalysts.^1,17^ CO_2_ treatment via the inverse Boudouard reaction employs carbon dioxide to selectively oxidize carbon deposits on the catalyst surface, successfully removing carbon, while protecting the metallic state of the catalyst. This method offers an alternative to traditional oxidative regeneration techniques, which—as mentioned above—often lead to sintering and loss of active surface area.^33^ Our research target is to highlight the effectiveness of CO_2_ treatments in regenerating Ni–Zr catalysts and their improved performance over consecutive DRM cycles. Generally, cyclic testing is employed in this study to simulate industrial operation and regeneration conditions, where catalysts are subjected to periodic decoking cycles to maintain performance and prevent irreversible deactivation.^34^ In practical industrial processes, particularly in dry reforming of methane (DRM), catalyst regeneration is frequently required to mitigate carbon deposition and sintering that can occur during prolonged operation. By conducting cyclic testing, we aim to mimic these real-world conditions and to evaluate the robustness of the catalyst especially under repeated reaction-regeneration cycles. This approach provides both valuable insights into the long-term stability and the reusability of the catalyst, which are critical for its practical applicability in industrial settings.

Structural and morphological analysis of the DRM- and CO_2_-induced changes of the Ni–Zr catalyst was obtained using a combination of surface- (X-ray photoelectron spectroscopy, XPS) and bulk structure- and chemical characterization methods (Scanning Electron Microscopy (SEM) and energy-dispersive X-ray spectroscopy (EDX)).

## 2 Methods and Materials

### 2.1 Catalyst Preparation

The intermetallic Ni–Zr sample was prepared by physical vapor deposition (PVD), utilizing small pieces of pure nickel wire (high purity, 1 mm thickness) and zirconium foil (Alpha Aesar, 99.5% purity, 0.1 mm thickness) under high vacuum conditions (1 × 10^–6^ mbar). Before deposition, the Zr foil was cleaned mechanically to remove oxide layers. The deposition of thin Ni films on Zr was carried out via thermal evaporation using a highly adaptable modular high-vacuum chamber. The chamber is based on a Schott Duran flat flange system with glass recipients.^35^ The setup includes home-built stainless steel ring modules compatible with standard glass flat flanges from Schott DN100 (inner diameter: 90 mm). Two metal rings, separated by fluorocarbon O-rings (diameter: 99 mm) and sealed with high-vacuum grease, were used instead of multiple inspection windows to maintain simplicity, utilizing a glass recipient. A quartz crystal microbalance with QS 008 Ag quartz crystals (Umicore) at 5 MHz resonance frequency monitors the coating thickness of the thin film. A home-built substrate mount holds the Zr foil substrate (18 × 20 mm^2^). The tungsten boat for thermal-resistive heating is positioned below the substrate mount. A PKR 361 Pirani/cold cathode gauge (Pfeiffer Vacuum AG, Aßlar, Germany) is used for pressure monitoring. The chamber is pumped by a turbo-molecular pump (300 L s^–1^) and a two-stage rotary vane vacuum pump, maintaining a base pressure in the low 10^–7^ mbar regime. Electrical feedthroughs connect the substrate mount and the boat suspension to a low-voltage transformer. During deposition, the substrate mount was heated to 170 °C, enhancing the adhesion and growth properties of the film. The entire setup, including the quartz crystal microbalance, boat suspension, and transformer connectors, is water-cooled.

**Figure 1:**
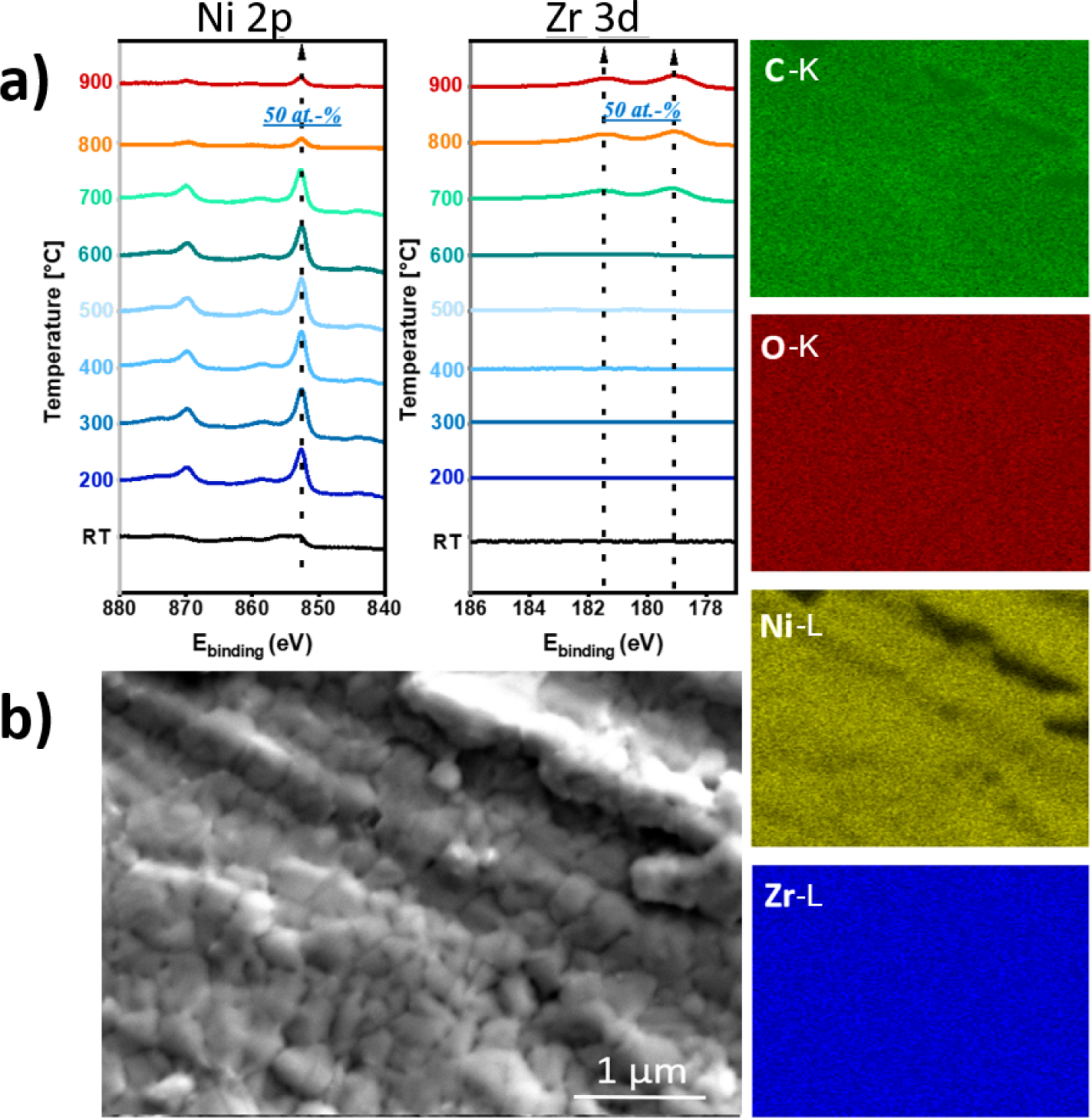
Panel a) Surface chemical characterization of the initial 100 nm Ni-on-Zr state as monitored during the annealing period by XPS between 25 and 900 °C. Panel b) Secondary electron contrast SEM image and EDX maps of the alloyed Ni–Zr surface after annealing to 900 °C, before DRM.

Before the DRM experiments, the Ni-coated surface of the Zr foil was transformed into a Ni–Zr bimetallic alloy layer within the UHV XPS/LEIS/AES chamber detailed in section 2.2. The cleaning process and subsequent alloy formation involved Ar^+^ sputtering (5 × 10^–5^ mbar Ar, 2 keV, 1 μA sample current) and gradual thermal annealing from 25 to 800 °C under UHV conditions (5 × 10^–10^ mbar), respectively. This treatment continued until XPS spectra indicated a saturated final alloy surface composition of ∼50 at % Ni and ∼50 at % Zr as the target catalyst composition for DRM application (Figure 1). An ultraclean Ni foil (Alfa Aesar, 99.994% purity, 0.1 mm thickness, 18 × 20 mm^2^) was used as a reference catalyst. The surface cleaning process for the Ni foil followed the same steps as for the Ni–Zr foil.

### 2.2 UHV System with Attached High-Pressure Batch Reaction Cell

The UHV system, equipped with an all-quartz recirculating batch reactor, is thoroughly detailed in ref (36) and engineered for quantitative catalytic and kinetic studies up to 1 bar on polycrystalline foils, with product detection either by continuous online MS analysis (HP GC-MS System G1800A) through a capillary leak, or by intermittent column injection GC-MS analysis. MS signals of CH_4_, CO and CO_2_ (*m*/*z* 15 + 16, 28 and 44) were externally calibrated and corrected for fragmentation. All DRM reactions were performed with initial partial pressures of 50 mbar CH_4_ and 50 mbar CO_2_. The reaction cell was backfilled with pure He to a total pressure of 1013 mbar to ensure efficient gas intermixing through recirculation and to enhance heat transfer to the sample by improving thermal conductivity. For the DRM tests, the reactor was heated at a constant rate of 10 °C min^–1^ to a final temperature of 800 °C, with an isothermal section for 30 min.

For quantification of the catalytic profiles, we have accordingly calculated the Turnover Number (TON) and Turnover Frequency (TOF) for the pure Ni sample as a reference and for the Ni/Zr catalyst based on CO TPD results and batch reactor data. To determine the number of Ni sites, we performed CO adsorption at 25 °C and according to temperature-programmed desorption (TPD) on both samples. Detailed calculations and explanations, along with the TPD profiles, are provided in the Supporting Information in the context of Figure S3. The average TOF value of ∼5 s^–1^ per Ni surface atom indicates highly efficient catalyst turnover under batch reactor conditions. Maximum TOF values were extracted from the individual inflection points of the TON vs time curves’ first derivatives and summarized in Table S1.

### 2.3 Scanning Electron Microscopy (SEM)

Scanning electron microscopy and corresponding energy-dispersive X-ray spectroscopy (EDXS) measurements were performed on a field-free analytical TESCAN Clara ultrahigh-resolution scanning electron microscope operated at 10 kV. EDX maps were collected using an Oxford Ultim Max 65 mm^2^ detector.

### 2.4 X-Ray Photoelectron Spectroscopy (XPS)

All XP spectra were collected using a Thermo Fisher Alpha 110 hemispherical analyzer and SPECS XR 50 twin Mg/Al Kα X-ray gun, attached to the UHV chamber. Mg Kα radiation was used in all experiments. All XPS data were analyzed using the CasaXPS software program, version 2.3.25 PR1.0. For peak fitting, a Shirley background was applied to all spectra. Deconvolution of all Zr 3d spectra included the metallic Zr^0^ component at a binding energy (BE) of 179.1 eV and ZrO_2_ at a BE of 183.0 eV.^37,38^ The Ni 2p_3/2_ peak is assigned a binding energy (BE) of 852.8 eV for the metallic component, while the C 1s BE values are 284.4 eV for graphitic carbon and approximately 282.2 eV for carbide-type carbon.^39^ To fit the O 1s spectrum, three different components were considered: ZrO_2_ at 530.2 eV, Zr suboxide at 531.2 eV and hydroxylated zirconium species at 531.4 eV.^40^

## 3 Results and Discussion

### 3.1 Formation of the Ni–Zr Intermixed Alloy State

Figure 1a displays the Ni 2p and Zr 3d XP spectra obtained during stepwise thermal annealing of the 100 nm Ni-coated initial state (25 to 900 °C). The Ni 2p spectra (left) show a decrease in the intensity of the Ni peak with increasing temperature. This suggests that Ni diffuses into Zr, indicating the formation of an alloyed state. Simultaneously, the Zr 3d peaks (right) increase, suggesting that the surface becomes Zr enriched due to metal intermixing. This transformation continues until the atomic percentage ratio between Ni and Zr reaches ∼43 at %: 43 at % (Table 1) at approximately 800 °C. These observations prove that highly dynamic surface transformations occur on the Ni–Zr catalyst during high-temperature annealing, even in the absence of a reactive gas environment. As only minor compositional changes were observed between 800 and 900 °C, the 900 °C-annealed state was assumed to be thermally stable with respect to ongoing metal interdiffusion during the subsequent DRM cycles, which did not exceed 800 °C maximum temperature.

**Table 1:** Overview of the Compositional Changes of the Ni–Zr Catalyst in Atomic Percentage (at) from the State before DRM to the 4th DRM Post-regeneration Cycle

Cycles/Treatments	Ni 2p (at %)	O 1s (at %)	C 1s (at %)	Zr 3d (at %)
**before DRM**	43.4	11.3	1.40	43.7
**1st DRM**	5.20	24.8	43.7	26.3
**2nd DRM**	4.40	23.0	46.8	25.8
**3rd DRM**	4.10	20.9	51.4	23.6
**4th DRM**	3.60	18.3	55.1	23.0
**after CO**_**2**_**treatment**	29.5	31.2	2.10	37.2
**1st DRM post-regeneration**	28.3	23.1	18.8	29.8
**2nd DRM post-regeneration**	28.0	22.0	22.6	27.4
**3rd DRM post-regeneration**	27.1	21.8	24.5	26.6
**4th DRM post-regeneration**	26.9	20.3	26.4	26.4

Figure 1b displays the complementary SEM image and EDX maps of the catalyst surface/bulk after annealing to 900 °C under vacuum conditions (p ∼ 5 × 10^–9^ mbar), showing the uniform distribution of Ni and Zr. The bimetallic state structurally manifests itself by coating the grinding-induced surface landscape with bubble-shaped bimetallic extrusions. In order to elucidate the role of the initial Ni layer thickness, we performed otherwise identical experiments for two nominal thicknesses of 100 and 500 nm Ni on Zr, yielding very similar results both with respect to structural and compositional changes (Figure S1 shows the analogous XPS characterization of the 500 nm sample as in 1a during thermal annealing) and catalytic performance. Therefore, we focus mainly on the representative results obtained on the 100 nm sample in the following.

### 3.2 Catalytic Dry Reforming of Methane Activity Before and After CO_2_ Treatment

**Figure 2:**
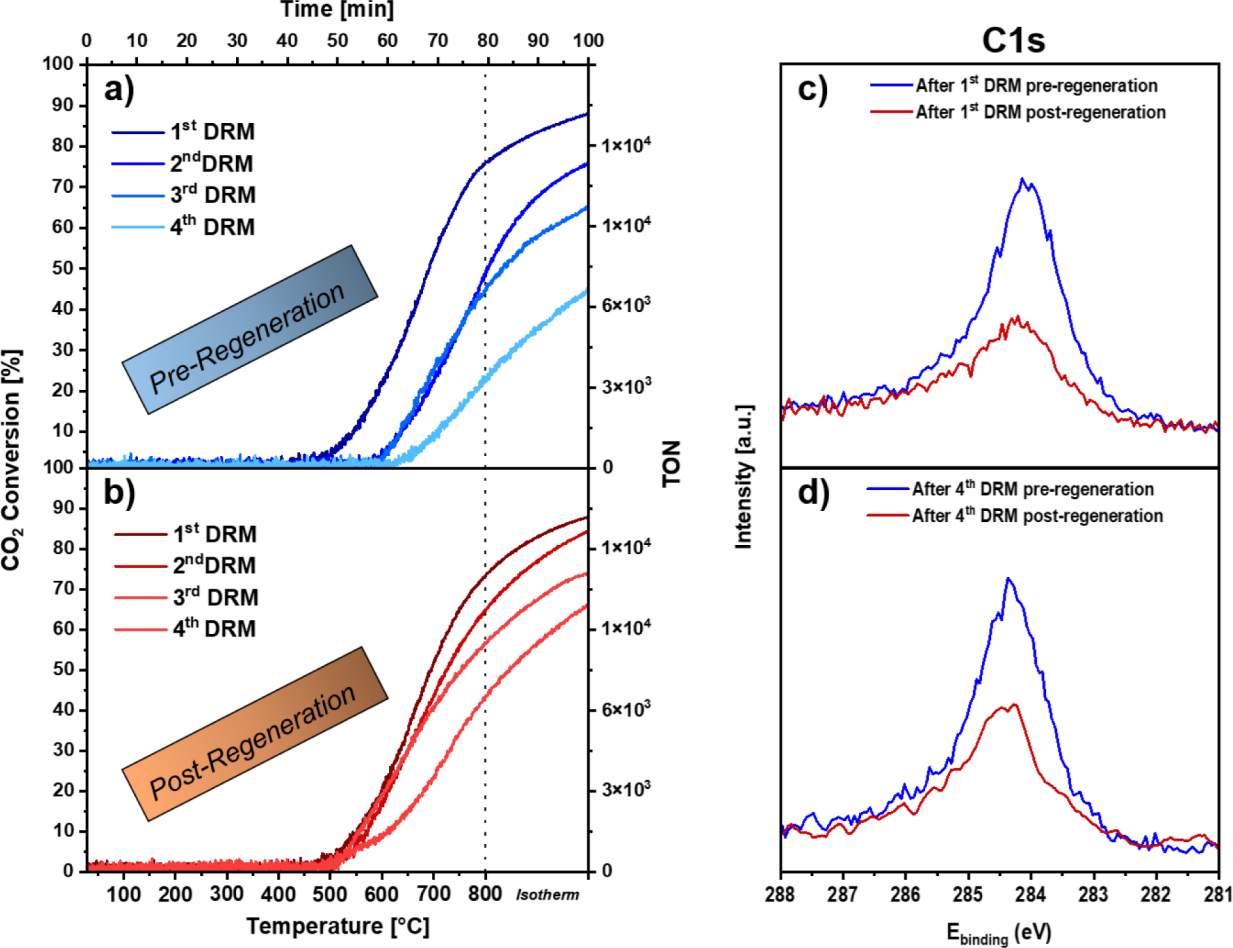
CO_2_ conversion profiles/TON numbers of the 1st, 2nd, 3rd, and 4th DRM cycle on the 100 nm Ni/Zr intermetallic catalyst. Panel a) DRM series before CO_2_ treatment; Panel b) after the CO_2_ treatment. Panels c) and d) C 1s XP spectra comparing the amount of carbon deposition before and after regeneration: Panel c) after the respective 1st DRM cycles; Panel d) after the respective 4th DRM cycles.

Quantitative temperature-programmed DRM experiments on the 100 nm Ni/Zr sample and a pure Ni foil as a reference (Figure S2) were performed in the UHV-compatible recirculating batch reactor. The pristine Ni–Zr sample in its initial intermetallic precatalyst state was tested with respect to its catalytic DRM performance in four consecutive DRM cycles (Figure 2a). Thereafter, the catalyst underwent an intermediate treatment in clean CO_2_ to remove carbon deposits. Finally, the regenerated catalyst was reevaluated in four additional DRM cycles (Figure 2b). This consecutive testing aimed at determining the catalyst performance and stability both before and after the CO_2_ regeneration. As illustrated in Figure 2a, the CO_2_ conversion profiles of the 100 nm Ni–Zr sample for the first–fourth DRM cycle before CO_2_ titration indicate a clear trend of decreasing catalytic activity with each successive cycle. The first DRM cycle shows the highest CO_2_ conversion slightly below 90%, indicating optimum initial activity. However, during the first four cycles, the maximum conversion decreases gradually to 45%. This decline is primarily attributed to progressive carbon deposition and sintering of Ni particles on the catalyst surface, which blocks and diminishes active sites and reduces the catalyst performance. However, the post-CO_2_ conversion profiles shown in Figure 2b indicate a notable improvement in catalytic performance. The first DRM cycle after regeneration exhibits high CO_2_ conversion, similar to the initial activity. The following cycles (2nd–fourth) also exhibit higher final conversions than their preregeneration counterparts, indicating not only successful regeneration but improvement in catalytic activity after each individual cycle preto-post CO_2_ treatment. The fourth postregeneration cycle, although not as high as the first cycle, shows a significant recovery, still reaching around 70% maximum conversion. This improvement demonstrates that CO_2_ treatments effectively remove carbon deposits, restore the initial catalytic performance and establish an enhanced degree of coking resilience. This trend is directly reflected by the XPS spectra in Figure 2c, comparing the composition of the catalyst surface before and after regeneration for the respective first DRM cycles. A significant reduction in the postregeneration carbon content, as expected, is evident. The intensity of the carbon peak (∼284 eV) is markedly lower after CO_2_ titration, indicating the successful removal of carbon deposits. The reduction of carbon intensity confirms the effectiveness of CO_2_ treatment for cleaning the catalyst surface and regaining active metallic Ni sites. Similar to Figure 2c, the XP spectra in Figure 2d show a decrease in postregeneration carbon intensity upon comparing the respective fourth DRM cycles. The carbon peak is again significantly reduced, highlighting diminished coking thanks to the intermediate CO_2_ treatment even after several cycles.

In summary, the XP spectra indicate that even after multiple DRM cycles, CO_2_ titration remains effective in protecting the catalyst surface by reducing accumulated carbon. Generally, the CO_2_ conversions on both the 100 and 500 nm samples exhibit a significant improvement, as compared to their respective preregeneration states (Figure S4). This noticeable recovery highlights the effectiveness of the regeneration process, not only by restoring the initial catalytic activity of the catalyst but by inducing an additional long-term anticoking effect. We note that the effectiveness of the CO_2_ treatment for regenerating the catalyst is not significantly affected by the initial Ni deposition thickness. Both 100 and 500 nm Ni–Zr intermetallic samples exhibit comparable improvements in CO_2_ conversion after CO_2_ titration, indicating that the regeneration process is robust across different Ni film thicknesses (the CO_2_ conversion profiles of a sample with 500 nm initial Ni film thickness are represented in Figure S4).

The data in Table 1 provide a comprehensive overview of the changes of the Ni, Zr, C, and O content (in at %) within the catalyst surface after the four initial DRM cycles, after the intermediate regeneration treatment in pure CO_2_, and after the four postregeneration DRM cycles. Before the first cycle, the as-grown bimetallic surface state displays equal amounts of metallic Ni (43.4 at %) and mostly metallic Zr (43.7 at %, Figure S5), with little carbon (∼1.4 at %). After the first DRM cycle, a considerable reduction of the Ni content to 5.2 at % was observed, along with a significant increase in carbon to ∼43.7 at %. This pronounced carbon deposition covers preferentially the active Ni sites, leading to associated catalyst deactivation (maximum CO_2_ conversion data see Figure S6). The Ni content further decreases to 3.6 at % after the fourth cycle, while the carbon content increases up to 55.1 at %. A clear correlation between increasing Ni coking and lowering the integral catalyst activity is evident.

The CO_2_ treatment performed after the fourth cycle significantly changes the catalyst surface composition. The Ni content increases to 29.5 at %, while the carbon content decreases to 2.1 at %, indicating the effective removal of carbon deposits and confirming the efficiency of CO_2_ treatments with respect to decoking. Past the first DRM cycle after CO_2_-regeneration, the Ni content remains high at 28.3 at % and the carbon content is significantly lower as compared to the state after the first DRM cycle before regeneration (18.8 at % vs 43.7 at %). This difference highlights that the CO_2_ treatment did not only remove a major part of the carbon deposits, but also helped to establish a more coking-resilient catalyst state. After the second postregeneration DRM cycle, the Ni content remained relatively stable (∼27 at %), indicating that the catalyst tends to retain its improved coking resistance. Despite a minor increase in carbon content from 22.6 at % after the second cycle, to 26.4 at % after the fourth cycle, coking remained significantly suppressed as compared to the preregeneration cycles. The relatively constant Zr content of around 27 at % observed during these cycles indicates that the compositional integrity of the catalyst is largely retained.

These strong indications of the 2-fold effectiveness of intermediate CO_2_ treatments for decoking and for enhancing the catalyst stability and durability highlighted the need to scrutinize the underlying structural effects.

### 3.3 Structure–Surface Composition–DRM Activity Correlation

**Figure 3:**
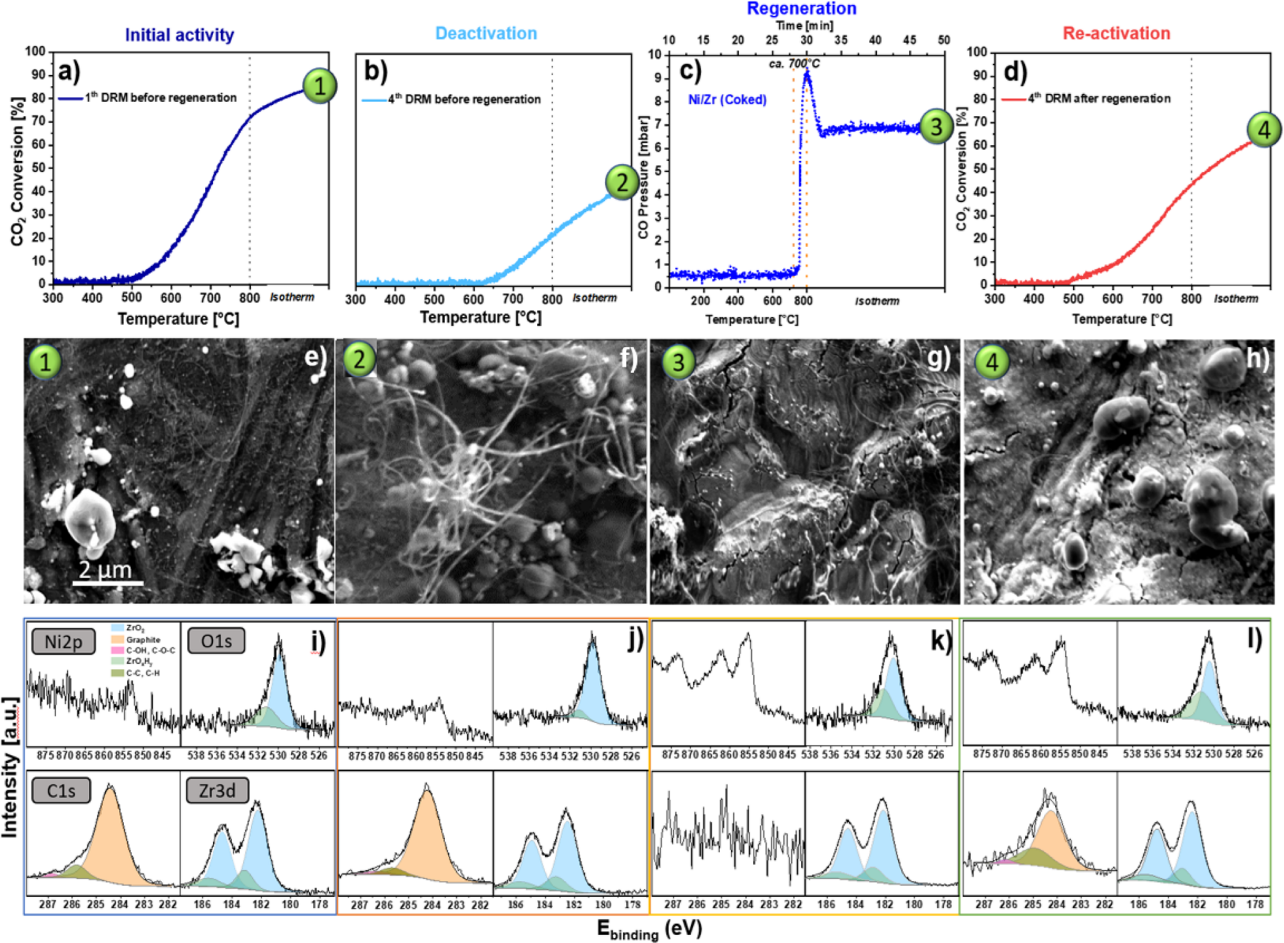
Structure–surface composition–DRM activity correlation on the 100 nm Ni–Zr bimetallic catalyst. Panel a) Initial activity: CO_2_ conversion profile of the 1st DRM cycle; Panel b) Deactivation: CO_2_ conversion profile of the 4th DRM cycle (corresponding to maximum deactivation); Panel c) Regeneration: CO pressure change in the batch reactor during titration in initially 100 mbar CO_2_ (heating rate ∼40 °C min^–1^); Panel d) Reactivation: CO_2_ conversion profile of the 4th DRM cycle after regeneration; Panels e)–-h) SEM images taken after cycles a)–d); Panels i–-l) overview of the Ni 2p, O 1s, C 1s, and Zr 3d XPS regions corresponding to the catalyst states indicated by the numbers (1)–(4). The XP spectra of the bimetallic state before the first DRM cycle, and of the respective catalyst states after the 2nd and 3rd preregeneration DRM cycles are provided in Figure S5.

**Figure 4:**
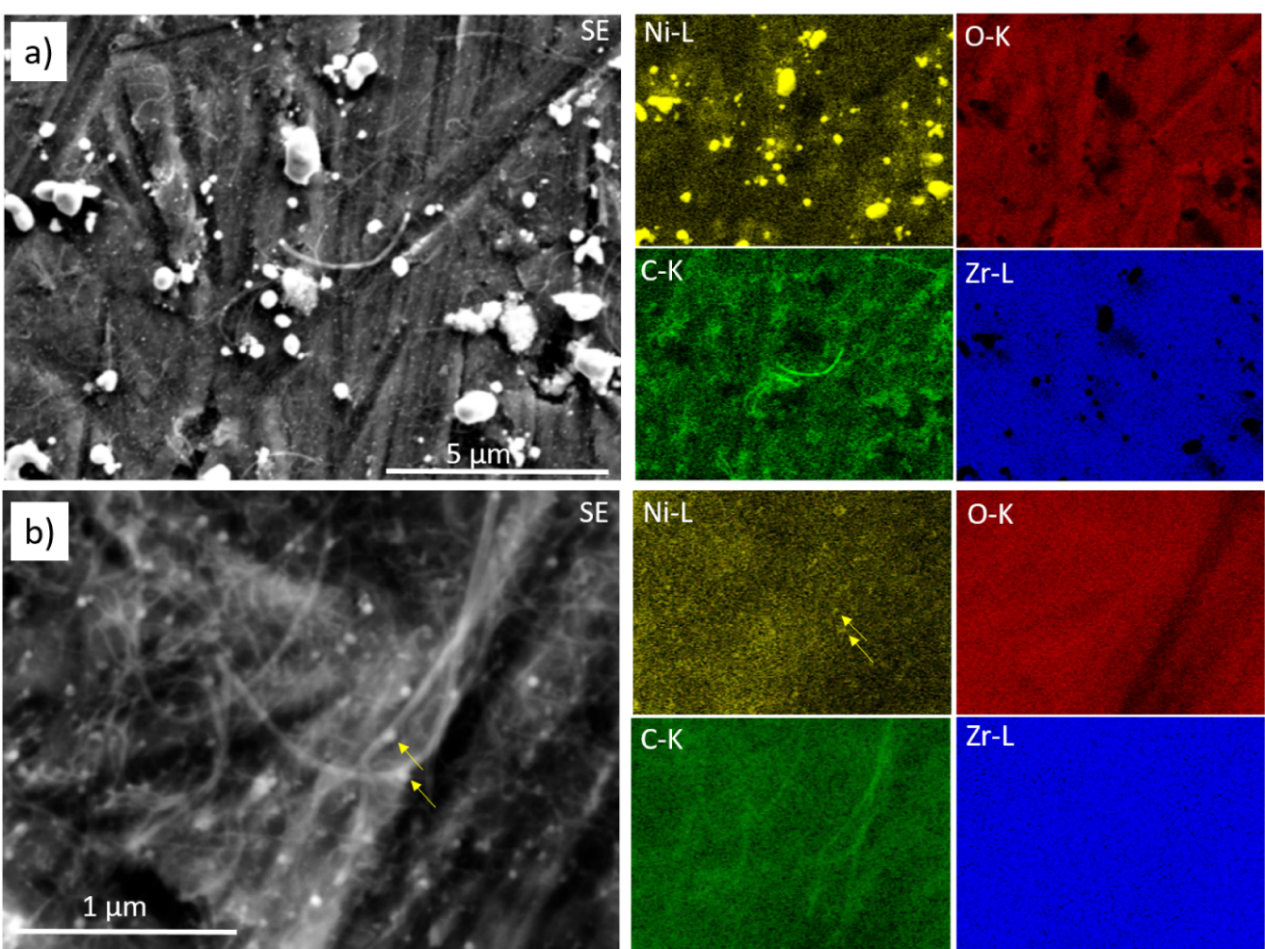
SEM and EDX characterization of the activated Ni–Zr catalyst surface after the 1st DRM cycle before CO_2_ regeneration. Panel a) Top row: secondary electron contrast of surface region with the agglomeration of μm-sized Ni particles, alongside the respective EDX maps of the Ni–L, O–K, C–K, and Zr–L edges. Panel b) Bottom row: The surface region with abundant nm-sized Ni particles and substantial coking by carbon filament formation. Yellow arrows have marked two Ni particles.

A comprehensive correlation of the changes in catalytic activity and the structure of the catalyst surface throughout the different stages of the DRM process and CO_2_ regeneration is provided in Figure 3, Panels a)-l). Panels a)-d) show the corresponding CO_2_ reaction profiles, Panels e)-h) the corresponding SEM images at selected points marked in green from (1) to (4), and Panels (i)-l) the XP spectra as a marker of the surface chemical state. A clear structure-surface composition-DRM activity correlation can be directly established. Panel a) shows the CO_2_ conversion profile during the first DRM cycle, highlighting the high initial activity without carbon deposits. The conversion starts to increase with a temperature slightly below ∼500 °C, reaches a turning point at ∼700 °C, and continues to increase toward ∼87% after reaching 800 °C. Panel e) shows the related SEM image obtained after the initial DRM cycle. Carbon contamination in the form of carbon filaments is visible, and during the in situ treatment in the DRM atmosphere, a large number of Ni nanoparticles on ZrO_2_ is formed. A dual size distribution is prevalent: we observe nm-sized Ni particles (size ∼30 nm), but some regions also exhibit very large, heavily sintered Ni domains (in the μm-size range). This situation is shown in more detail in Figure 4. The top row and bottom row display regions with larger and smaller Ni particles, respectively, on different scales to visualize the respective particle sizes. In the lower left panel, two yellow arrows point out two small Ni particles.

**Figure 5:**
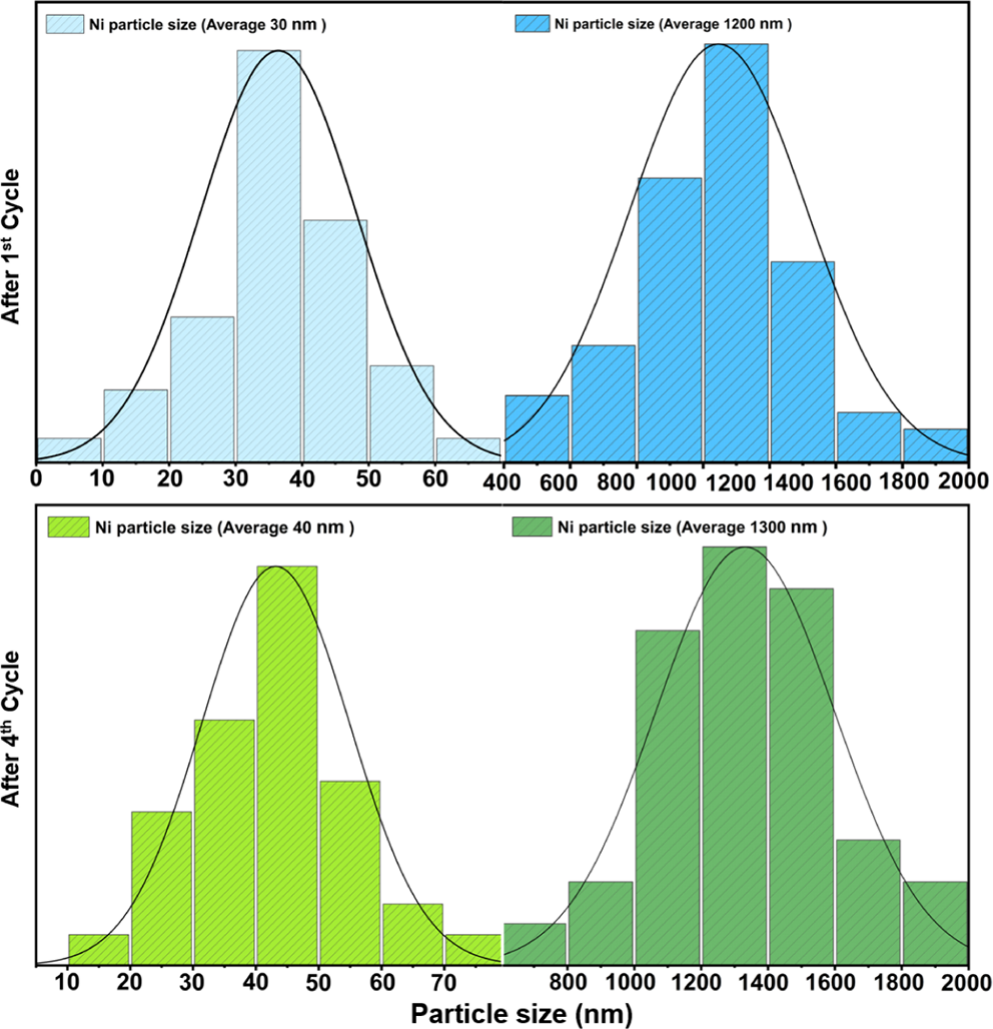
Particle size distributions of Ni nanoparticles before CO_2_ regeneration after the 1st cycle (top) and 4th cycle (bottom) DRM cycle.

For a better understanding of the evolution of Ni particle size after several DRM cycles, Figure 5 illustrates the size distribution of Ni particles for the catalyst at two distinct stages before CO_2_ regeneration, i.e., after the first DRM and after the fourth DRM cycle. For each histogram, we have evaluated >100 particles in multiple SEM images to provide reliable statistics. The common denominator of both stages is the bimodal size distribution: average Ni particle sizes of around 30 nm–40 nm, as well as between 1200 and 1300 nm, are observed. Slight sintering of the Ni particles between the first and fourth DRM cycle is evident by the shift in the Ni particle sizes.

**Figure 6:**
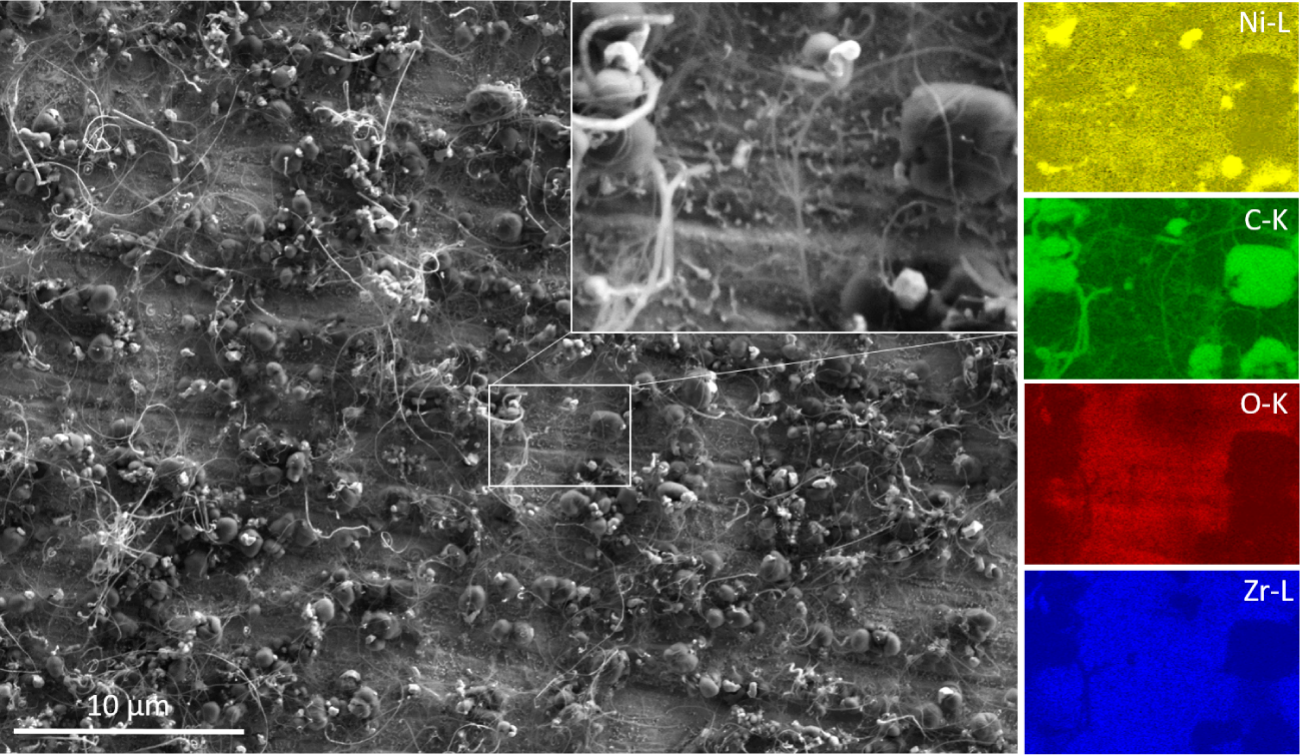
Secondary electron contrast SEM image and EDX characterization of the Ni–Zr catalyst after the 4th DRM cycle before CO_2_ regeneration. EDX maps of the Ni–L, O–K, C–K, and Zr–L edges are shown in the right column.

The presence of carbon filaments across the surface indicates that carbon deposition on top of especially the larger Ni domains results from the DRM process. Coking is already quite strong after the first DRM cycle on the “pre-CO_2_” sample according to Table 1, and becomes gradually enhanced from this initially high level toward a more and more carbon-rich and deactivated state. Apart from the difference in Ni particle size, another main difference between the two regions is the much lower number of carbon filaments in regions with smaller Ni particles. Both Figure 3f and Figure 6 indicate that especially the larger Ni particles act as a source of the growth of carbon filaments. This can be seen both in the bottom left SEM image and in the respective carbon EDX maps.

The respective EDX panels corroborate the exact location of these particles. The related XP spectra in panel (i) (Figure 3) show the Ni 2p, O 1s, C 1s, and Zr 3d regions after the initial DRM cycle, indicating a relatively low Ni 2p intensity due to pronounced shielding of the Ni metal domains/particles with reaction-induced carbon deposits. The O 1s intensity increases considerably, which is due to the oxidative decomposition of the Ni–Zr alloy, forming a surface-bound ZrO_2_ layer. The respective Zr 3d intensity decreases, due to the shielding of the extended carbon filaments covering vast regions of the surface (cf. Figure 4, bottom left image, counterbalancing the increase due to the surface oxidation).

In panel b) the CO_2_ conversion profile during the fourth DRM cycle is shown. The reaction onset temperature is shifted to ∼620 °C, and the maximum conversion of ∼40% reached at the end of the isothermal period is significantly lower than after the initial cycle (∼87%), indicating pronounced deactivation due to carbon deposition (i.e., coking) and sintering of the Ni particles. Panel f) and Figure 6 show the surface structure as imaged by SEM after the fourth DRM cycle. Two features are notable: the number of 1D-filament-like carbon deposits and coating graphitic carbon layers increased considerably, which is accompanied by a further increase in number of the large Ni particles. The presence of Ni-covering 2D-carbon deposits is rather visible in the EDX images, specifically on top of the large Ni domains (upper right, zoomed area, and related Ni-L and C–K maps). Altogether, the catalyst exhibits extreme coking and high-grade encapsulation of the Ni particles by carbon. Not surprisingly, this catalyst state represents the most deactivated one with respect to all other performed DRM cycles. Nevertheless, the coke deposits still block only a part of the active Ni sites, as the residual activity suffices to achieve a maximum conversion of ∼45%.

The related XP spectra, obtained after the fourth DRM cycle (Panel j), exhibit a similar degree of selective Ni coking as after the first DRM cycle, indicating that the major deactivation effect by coking occurs already during the first DRM cycle, with only a slight increase in C 1s intensity from ∼44 at % to around 55 at % in the subsequent cycles.

Whether the 1-dimensional (1D) or 2-dimensional (2D) carbon deposits, respectively,^40^ cause the main deactivating effect, is rather obvious. 2D carbon structures are more planar and cage-like and block a large fraction of the available Ni surface sites. As shown in a previous study,^1^ the main reason for the deactivation of the active catalytic sites mainly located at the Ni-ZrO_2_ phase boundary is 2D-carbon, which encapsulates preferentially the larger Ni catalyst particles. On the other hand, local 1-dimensional (1D) filament formation on some anchored particles is less detrimental to catalytic performance than encapsulating carbon, as it does not block active sites directly. The larger particles exhibit a smaller relative contribution of Ni-ZrO_2_ interfacial sites and require long-range diffusion of carbon toward the active metal-oxide interface sites for carbon clean-off by reaction with CO_2_. Therefore, they are prone to much stronger coking (and vice versa, much more sluggish decoking) than Ni nanoparticles with a relatively large metal-oxide phase boundary contribution and short diffusion pathways of carbon toward the phase boundary.

**Figure 7:**
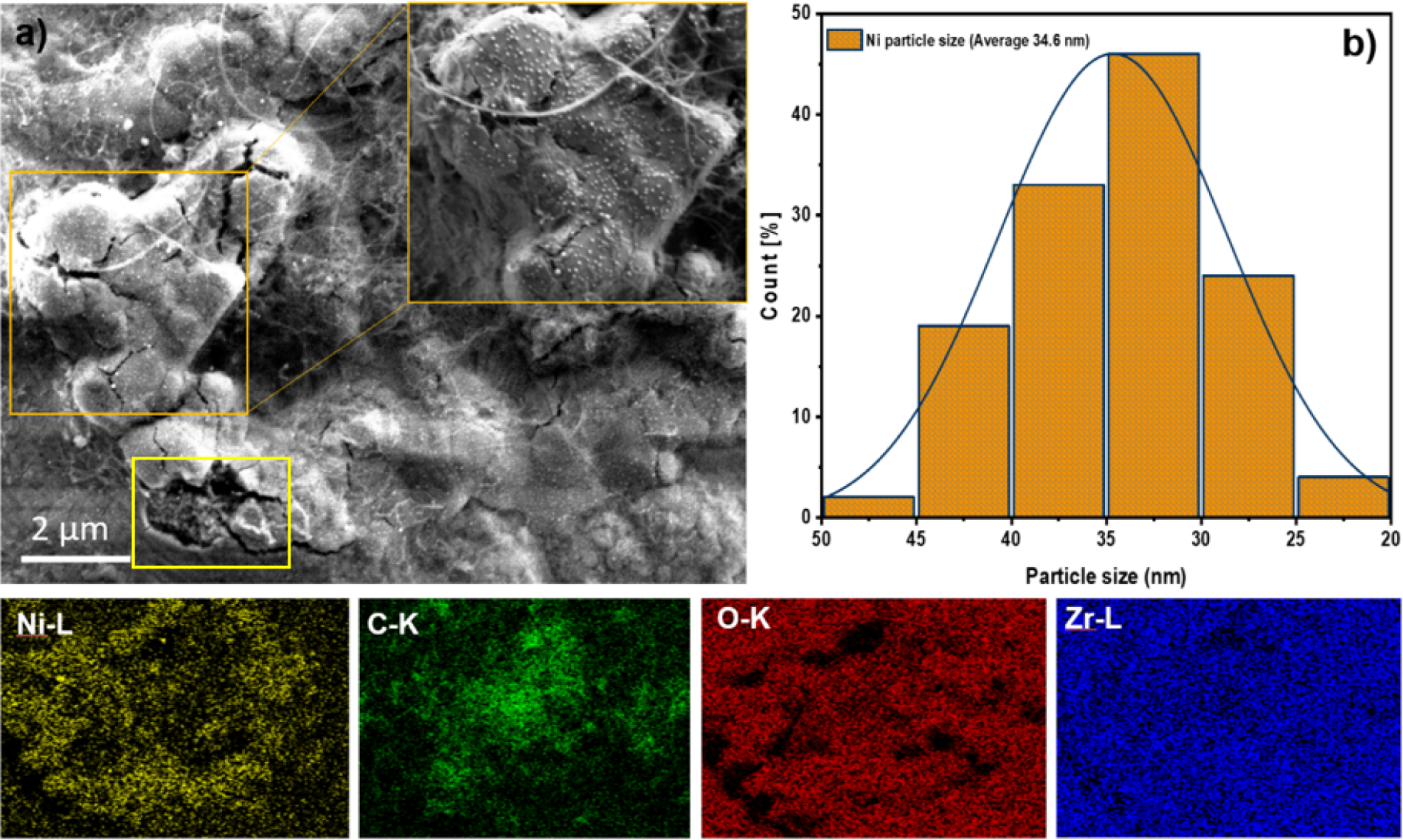
State of the initially coked Ni–Zr catalyst surface after regeneration in CO_2_. Panel a) SEM and EDX images (bottom row). Panel b) shows the respective Ni particle size histogram. The yellow frame in Panel a) shows the region, where a foam-like Ni-enriched particle is found beneath a cracked ZrO_2_-covered region. An area with an array of well-ordered Ni particles is magnified in the orange frame. The EDX maps correspond to the image in Panel a).

After the first four DRM cycles and the associated coke accumulation on the catalyst surface, CO_2_ titration of the deposited carbon was tested as a promising technique for coke removal (Panel c). The CO_2_ regeneration experiment was performed in a pure 100 mbar CO_2_ atmosphere between room temperature and 800 °C at a heating rate of 40 °C min^–1^ in the high-pressure batch reactor cell. Above ∼700 °C, a rapid reaction onset was observed, and the final level of the formed reaction product CO was reached after ∼33 min, indicating effective carbon removal via the reverse Boudouard reaction. The SEM image shown in the associated Panel g), obtained after CO_2_ treatment demonstrates the removal of a large fraction of the carbon deposits, except for some persisting, unreactive filamentous carbon species, and well-dispersed Ni particles (cf. also Figure 7). The XP spectra of Panel k) show an almost 100% decrease in the carbon intensity and a complete recovery of the Ni 2p peak. Obviously, the few remaining carbon filaments do not contribute to a measurable C 1s intensity.

The fourth DRM cycle after CO_2_ titration is illustrated in Panel d. Thanks to the reactivation step in CO_2_, the maximum CO_2_ conversion amounts to almost 70%, in contrast to only ∼40% after the fourth cycle before the CO_2_ treatment, implying that not only successful catalyst regeneration and restoration of its catalytic activity was accomplished, but a more coking-stable state of the catalyst was obtained. The lower carbon content of 26.4 at % (as compared to 55.4 at % after the fourth pretitration DRM cycle, see Table 1) corroborates the improved coking resilience of the CO_2_-reactivated catalyst. The corresponding SEM image (Panel h) shows a comparatively clean catalyst surface with a relatively low amount of visible filamentous carbon species. The related XPS spectra in Panel l) show a reduction in carbon content as compared to the fourth preregeneration DRM cycle and an accordingly enhanced Ni 2p intensity, proving comparatively weaker Ni-shielding and thus, Ni coking.

### 3.4 Structural Consequences of CO_2_ Regeneration

To emphasize the surface structural consequences of CO_2_ regeneration, Figure 7a presents the SEM and EDX images of the Ni/Zr catalyst surface obtained after CO_2_ treatment. The images highlight the effectiveness of CO_2_ treatments for regenerating the catalyst, not only by removing a large part of the carbon deposits but—as will be shown in the following—also by enhancing the fraction of finely dispersed nickel nanoparticles at the surface.

In comparison to Figure 6, the surface appears significantly devoid of carbon filaments and encapsulating carbon structures, indicating highly effective decoking. The inset (orange frame) highlights regions on which uniformly dispersed uncoked Ni nanoparticles are visible, indicating that exposure to clean CO_2_ has successfully increased the Ni dispersion. In contrast to before CO_2_ regeneration, where the catalyst displayed a bimodal distribution of Ni particle sizes (Figure 5), following regeneration, but before a subsequent DRM cycle, the particles became more uniformly distributed (monomodal size distribution), with average sizes of approximately 35 nm, as illustrated in the histogram in Figure 7b. The images demonstrate that basically no large Ni domains are left at the surface, in stark contrast to the catalyst state before CO_2_ regeneration, which exhibits de facto a bimodal size distribution with very large Ni particles among a large number of much smaller particles. We note that the structural origin of this “in-situ” DRM-induced size distribution is not fully understood to date, but it is safe to say that the gas phase switches during a DRM cycle from a rather oxidizing state (CO_2_ + kinetically not activated CH_4_ at lower temperatures) to a much more strongly reducing state. Once significant partial pressures of the reaction products CO and H_2_ prevail in the batch reaction cell at high temperatures, oxidized forms of Ni will be reduced swiftly to the metallic state. The open question is whether correspondingly extended NiO domains are already present before DRM product formation sets in, or locally enhanced sintering leads to the formation of the large domains past the reductive switching of the gas phase upon DRM onset.

**Figure 8:**
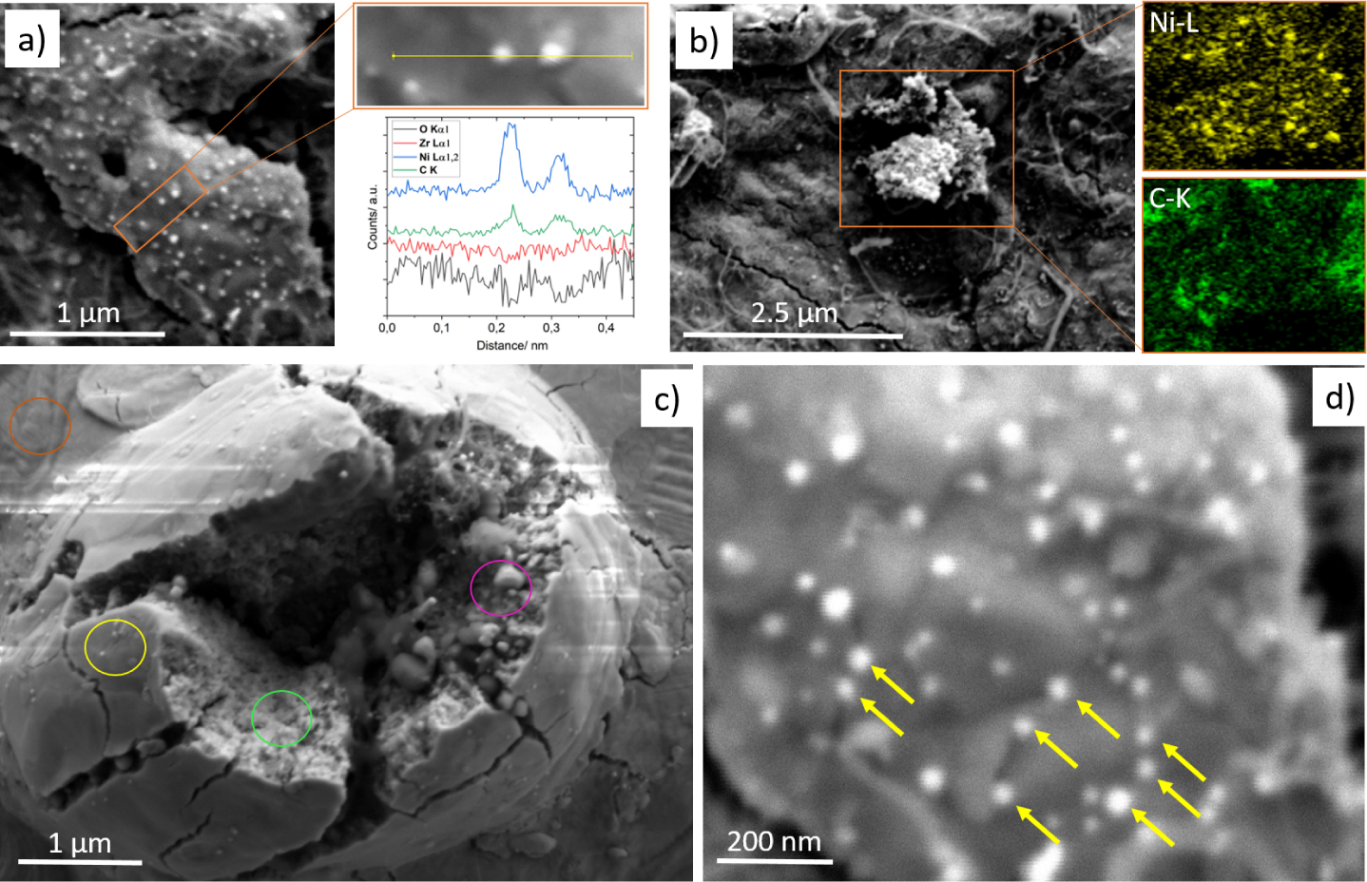
SEM and EDX images obtained after the 4th post-CO_2_ DRM cycle. Panel a) Persisting, evenly dispersed Ni nanoparticles and respective EDX line scan; Panel b) Foam-like Ni extrusion with locally reduced coke deposition; Panel c) Survey of a breakout region with chemically and structurally distinct regions marked by colored circles. The respective EDX spectra obtained within these regions are provided in Figure S7 (green: S7 (a); yellow: S7 (b); purple: S7 (c); orange: S7 (d)). Panel d) Enlarged region of Figure 8a, highlighting that a vast number of Ni particles (marked by yellow arrows) are found at cracks in the ZrO_2_ covering layer.

However, the absence of too large Ni particles and domains is very important for retaining high catalytic activity and for counteracting large-area coking.^1^ Their vanishing can be explained by ongoing corrosion of a remaining bimetallic reservoir below the coked Ni-ZrO_2_ top layer in CO_2_, leading to overgrowth by extra ZrO_2_ resulting from oxidation of bimetallic NiZr. The decoration of the newly formed ZrO_2_ top layer, which appears bulged and torn open (especially in regions where one suspects the covered large Ni domains), by evenly distributed small Ni particles is most likely caused by a special, yet hardly understood transport mechanism of hidden and/or buried forms of nickel toward outer surface regions. The lower left region of Figure 7a (yellow rectangle) allows for a view of the state of the catalyst underneath the CO_2_-induced ZrO_2_ top layer, as it is locally detached and the underlying material becomes visible. This material is rather rich in Ni (cf. Figure 8) and despite the fact that it originates to some extent from the large “monolithic” Ni domains located at the surface before CO_2_-regeneration, it consists of a rather finely dispersed, foam-like Ni-rich conglomerate. The observed average Ni particle size of ∼35 nm after regeneration is critical for catalytic performance due to the well-established relationship between particle size and catalytic activity in Ni-based systems. Smaller Ni particles offer a higher surface area-to-volume ratio, providing more accessible active sites for DRM operation. Additionally, smaller particles are more resistant to carbon filament formation, a common cause of deactivation in DRM processes. Studies indicate that Ni particles within the range of 10–50 nm exhibit optimal catalytic activity and coking resistance.^41^

An explanation for the foam-like forms of Ni can be given in the context of the course of the CO_2_ treatment shown in Figure 3c. Heating until ∼700 °C takes place in a pure CO_2_ atmosphere, but after the sudden onset of the reverse Boudouard reaction connected with the carbon deposits above 700 °C, an approximate 10:90 ratio of CO:CO_2_ is reached. This ratio drops quickly toward a final ∼7:93 ratio at ∼800 °C. Essentially, this means that a part of the already formed CO reacts back toward CO_2_ in the batch reaction cell, indicating partially reversible consumption of CO by the oxidized catalyst. This effect can explain the existence of the newly formed foam-like forms of Ni: until 700 °C, the large Ni domains underneath the visible cracks of the top layer are exposed to a rather strongly oxidizing gas environment (pure CO_2_ without CO-content, but O_2_ content of ≤10 ppm), which converts them at least partially to NiO.^43^ Upon the onset of decoking, the gas phase becomes at once much more reductive. According to the partial pressure- and temperature-dependent equilibrium reaction
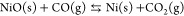


the stability limit of NiO is reached at a temperature of ∼700 °C in a 7:93 CO:CO_2_ gas environment. The temperature-dependent changes in Gibbs free energy (Δ*G*^0^_T_) and the equilibrium constants for the oxide reduction reaction were determined using the NIST series expansions for the standard enthalpy change (Δ*H*^0^_T_) and standard entropy change (Δ*S*^0^_T_) of CO_2_(g), CO(g), Ni(solid) and NiO(solid) and the above-mentioned CO:CO_2_ ratio, corresponding to partial pressures of 8.4 mbar CO and 91.6 mbar CO_2_ in the reactor. Details of the calculation are given in the Supporting Information. Therefore, already existing NiO is rereduced toward Ni, while the simultaneously reformed CO_2_ may act as an expanding agent within the cavities to form a foam-like state of Ni. The associated volume expansion can explain the widening of the cracks and the protruding breakout regions, which are visible all over the place in Figure 7a. As mentioned before, the exact formation mechanism of the evenly distributed Ni nanoparticles located on top of the ZrO_2_ shell remains to be studied, but it is clear that exactly these particles, and in particular their phase boundary to ZrO_2_, represent the most DRM-active and coking-resilient structural entities on the catalyst.^1^

As discussed above, the redispersion process during CO_2_ treatment involves a series of interconnected redox reactions, including the oxidation of Ni, oxidation of carbon deposits, and subsequent reduction of Ni. In the first step, Ni undergoes partial oxidation due to the oxidizing nature of the CO_2_ atmosphere.^42^ As the temperature increases (∼700 °C), the reverse Boudouard reaction sets in, reducing CO_2_ to CO and creating a reducing gas environment. In this CO/CO_2_ gas mixture, the reduction of oxidized Ni back to its metallic state becomes thermodynamically unavoidable.^43^ This sequence of redox reactions appears to promote the redistribution of metallic Ni particles. We note that our results resemble a similar redispersion process as described recently in ref (44). In this study, although related to carbon-encapsulated Ni particles within a Ni/C electrode, the presence of a carbon layer, which initially encapsulates metallic Ni particles, is beneficial with respect to oxidative Ni redispersion. As the oxidizing atmosphere penetrates the carbon shell, Ni is oxidized within the layer. The resulting Ni species (e.g., NiO_*x*_) are mobilized and forced to escape from the encapsulating layer due to limited space and volume expansion, which leads to their eventual deposition as smaller, more uniformly distributed NiO_*x*_ particles on the surface. Upon onset of the inverse Boudouard reaction, the still available carbon deposits are largely consumed and the environment shifts to a CO/CO_2_ mixture. This causes the oxidatively redispersed NiO_*x*_ species to become reduced back to metallic Ni.^45^ Our observed foam-like, Ni-rich structures after CO_2_ regeneration (Figure 8b) can also be interpreted as “markers” for enhanced Ni mobility, and we suggest that they also form during the above-described redox sequence due to the transformation of larger, strongly carbon-encapsulated Ni aggregates into surface-enriched intermediate shapes.

Because of the enhanced Ni mobility, the resulting uniform metallic Ni particle size distribution (∼35 nm) observed postregeneration is, therefore, at least partially caused by the oxidative breakdown of larger, encapsulated Ni domains and the subsequent, reductive stabilization in smaller, catalytically active metallic Ni forms.

Beyond redox redispersion, ongoing oxidation of the metallic Zr species below the catalytically active top layer leads to the exsolution of additional zirconia, which not only creates new nucleation sites for small Ni particles, but also leads to the overgrowth and encapsulation of larger Ni domains. The local interplay between encapsulation of the latter, redispersion of Ni, and formation of new ZrO_2_surface area contributes to the enhanced abundance of smaller Ni particles at the surface, at the cost of the large ones, as confirmed by SEM and particle size analysis.

The analysis of the catalyst after multiple DRM experiments, which will be discussed in the following, reveals why the regeneration process in pure CO_2_ is particularly effective. The SEM images and EDX elemental maps obtained after the fourth post-CO_2_ DRM cycle are presented in Figure 8, providing an in-depth analysis of the regenerated and 4 times DRM-exposed Ni–Zr catalyst surface. The topographic SEM image (Figure 8a) shows a catalyst surface with a large number of evenly dispersed nickel nanoparticles. The inset image (upper right) and the EDX line scan reveal detailed information about the elemental distribution along a specific line on the surface. The highlighted line scan data (yellow line) indicate the presence of nickel peaks within a ZrO_2_ environment, confirming the rather unchanged presence and the pronounced thermochemical stability of the finely dispersed nickel nanoparticles across the entire catalyst surface.

The line scan data of Figure 8a show distinct peaks for Ni, indicating a rather stable size, shape, and dispersion of the surface-located Ni nanoparticles during the four DRM cycles past CO_2_ treatment. The topographic image in Figure 8b highlights a cluster of foam-like Ni, with the inset showing the respective EDX maps for Ni and carbon. The respective C–K map shows only local carbon deposition, indicating an enhanced coking stability of these structures as compared to the bulk-like pre-CO_2_ Ni species, which may also contribute to the generally improved coking resilience of the postregeneration catalyst.

Figure 8c presents the SEM image of a typical CO_2_-induced breakout region of the Ni–Zr catalyst surface after the fourth post-CO_2_ DRM cycle. The respective EDX spectra obtained within the marked regions are shown in Panels a) to d) of Figure S7. Within the green-marked area located at a porous section of the inner surface region of the breakout crater, ZrO_2_ is predominant, whereas the Ni (5.4 at %) and the carbon content (14 at %) are relatively low. In the yellow-marked area situated at the outer surface of the shell, ZrO_2_ is even more abundant, the Ni content drops to only 1.6 at % and the carbon content to 8 at %, revealing the most coking-resilient surface region decorated by active Ni nanoparticles. In contrast, the purple-marked area contains larger, probably sintering-induced Ni domains. Although the amount of Ni is even a bit lower than that in the green region (4.9 at % vs 5.4 at %), the amount of carbon is much higher at ∼50.9 at %, once again highlighting the disadvantages of too large Ni particles or domains. The orange region, located outside the breakout protrusion, is entirely dominated by the ZrO_2_ signals. In summary, the data confirm that the foam-like Ni in the green-marked area is less prone to coking than the bulk-like Ni within the purple area, exhibiting almost four times the amount of carbon.

Based on the results of Figures 7 and 8, the advantages of regenerating the catalyst in pure CO_2_ can be summarized as follows:

(1) Larger, bulk-like nickel particles, which were abundant on the coked catalyst before regeneration and shown to exhibit strong and irreversible coking, become covered or encapsulated by a newly formed ZrO_2_ layer upon the regenerating CO_2_ titration.

(2) While heating in initially clean CO_2_, they undergo a redox cycle involving oxidation at lower, and reduction back to metallic Ni at higher temperatures, as a specific consequence of the used batch reactor in which CO (formed by the reverse Boudouard reaction) is accumulated. This redox cycle is accompanied by local CO_2_ gas evolution and induces porous and finely dispersed forms of Ni metal,^43^ which exhibit reduced coking in comparison to their parent bulk-like structures. Certain analogies to pore-forming processes toward Ni-YSZ cermet materials, which involve internal gas evolution and volume shrinking of the metallic phase toward a foam-like state, appear likely.

(3) The ongoing redox processes promote the formation of abundant, evenly size-distributed Ni nanoparticles with extended phase boundaries to the surrounding ZrO_2_, which resist coking much better. Although the detailed transport mechanism of Ni toward the outer surface of the ZrO_2_ shell is not yet understood, the redispersion of Ni nanoparticles through CO_2_ regeneration is an obvious result, which increases the number of active and coking-resilient sites and, therefore, the coking stability of the catalyst through multiple DRM cycles.

Taken together, these results clearly demonstrate the effectiveness of CO_2_ titration in regenerating and further protecting initially intermetallic Ni/Zr catalysts used for the dry reforming of methane (DRM). The CO_2_ conversion for any sample, regardless of deposition thickness, is significantly improved after regeneration, and the nickel particles are successfully redispersed across the catalyst surface. The size distribution of the Ni particles after the fourth post-CO_2_ DRM cycle (Figure S8) again shows an average size of ∼35 nm, resembling that directly after CO_2_ regeneration (Figure 7b) and after the first DRM cycle post-DRM treatment (Figure S9), and indicating the effectiveness of CO_2_ treatment in redistributing larger Ni particles. The conservation of the improved, uniform size distribution demonstrates the high resistance of postregeneration Ni particles to sintering and highlights the beneficial structural role of the CO_2_ treatment for maintaining catalytic stability and performance over multiple DRM cycles. As the catalyst undergoes in summary 400 min of high-temperature DRM treatment at 800 °C, it also underscores the midterm thermal particle stability.

In other words, thermal cycling, as commonly encountered in industrial processes such as dry reforming of methane (DRM), subjects catalysts to repeated heating and cooling, which can exacerbate sintering and the formation of large metal aggregates.^46^ These structural changes often lead to diminished active site availability and overall catalytic efficiency. In this study, CO_2_ treatment has demonstrated its ability to reverse some of these deleterious effects by redistributing and further stabilizing Ni particles, as evidenced by the uniform and sinter-resilient particle size distribution observed both directly after regeneration and after four further DRM cycles. On the other hand, the associated, highly efficient decoking reaction highlights the efficiency of the CO_2_ regeneration process in restoring a high number of active catalytic sites and maintaining high catalytic performance over several successive DRM cycles. Compared to traditional oxidative regeneration techniques, which often result in sintering and a loss of active surface area,^28^ CO_2_ regeneration avoids these issues by selectively oxidizing carbon deposits without compromising the dispersed metallic state of the catalyst due to deep oxidation, but even improving it. This method not only preserves the structural integrity of the Ni/Zr catalysts, but also ensures their long-term stability and efficiency during multiple cycles.

## 4 Conclusions

This study emphasizes the significant potential of CO_2_ regeneration as a strong and efficient regeneration technique for Ni–Zr catalysts used in DRM applications. The CO_2_ treatment strategy effectively addresses two major challenges associated with DRM catalysts: carbon deposition (coking) and nickel particle redispersion, thus avoiding loss of activity upon sintering. By removing and reducing the carbon deposits that accumulate on the catalyst surface during the DRM process, CO_2_ treatment reverts the coke-induced deactivation of the catalyst. The intermediate CO_2_ treatment reinforces rather than compromises the structural integrity of the catalyst. During CO_2_ treatment, the ongoing redox processes enhance Ni redispersion by suppressing larger sintered Ni domains and promoting the abundance of smaller, uniformly distributed particles (∼35 nm). This improves the availability of coking-resilient active sites and reduces susceptibility to sintering in subsequent DRM cycles. The ZrO_2_ support structure remains intact throughout the regeneration process, as proven by SEM and EDX mapping. There is no evidence of structural degradation or loss of integrity at the Ni-ZrO_2_ interface.

In addition, this method aids in redispersing the nickel in the form of surface-located nanoparticles, ensuring that they remain well-dispersed and accessible as active entities on the catalyst surface. The ability to regenerate Ni–Zr catalysts using a simple and cost-effective CO_2_ treatment significantly boosts their operational lifespan. CO_2_-treated Ni–Zr catalysts ensure a more stable and reliable conversion of methane and carbon dioxide into valuable synthesis gas by maintaining high catalytic activity over multiple cycles. Overall, the integration of a CO_2_ regeneration treatment into the DRM process not only increases catalyst performance but may also support wide economic and environmental goals, rendering it an important advancement in the field of sustainable chemical processes.

**Scheme 1:**
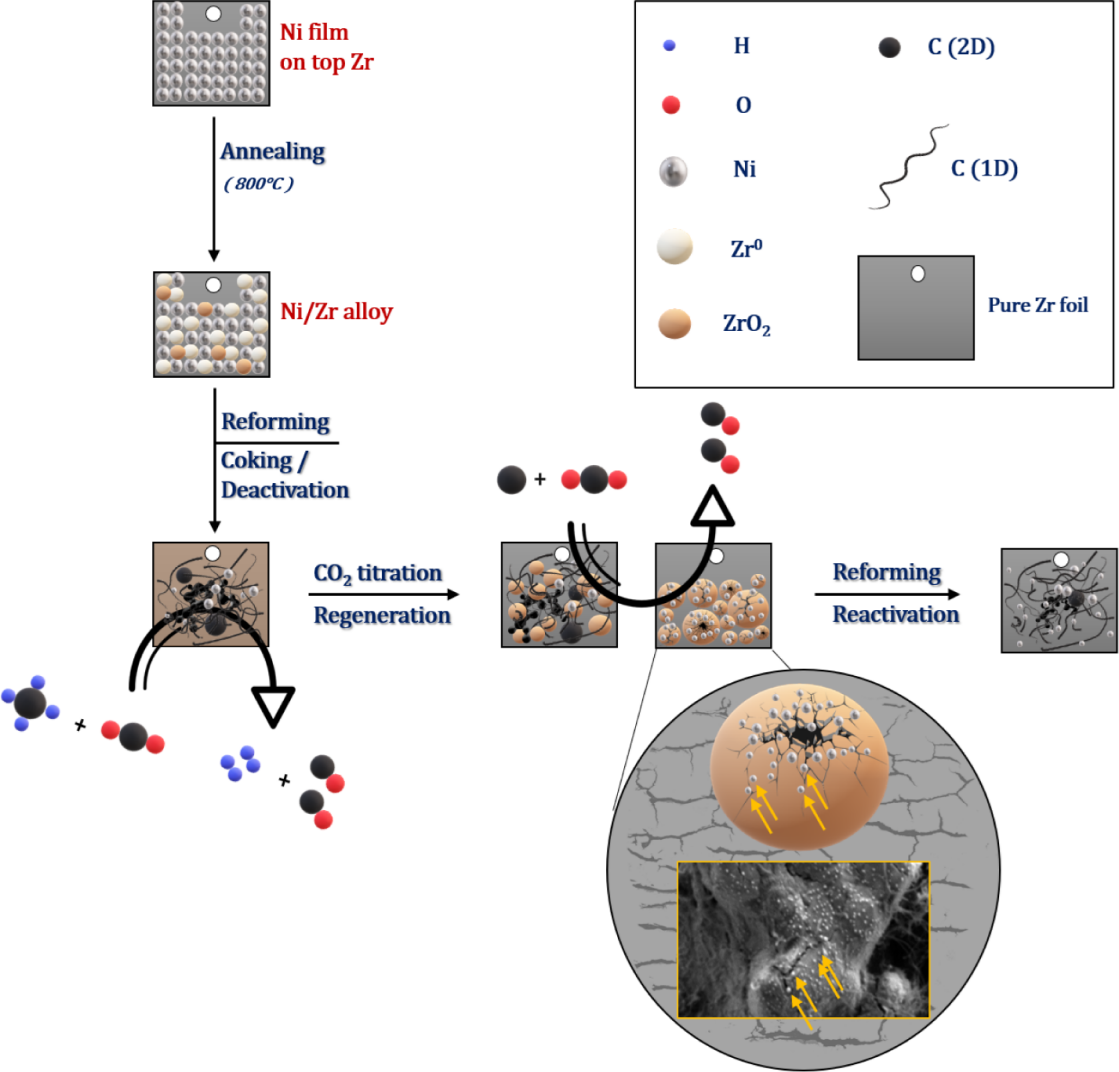
Proposed Ni–Zr Catalyst Activation/Regeneration/Re-activation Process for Initially Bimetallic Ni–Zr Dry Reforming of Methane Catalysts

The proposed mechanism for boosting the catalytic activity and inducing the associated structural changes on the surface is highlighted in Scheme 1.

## Supporting Information Available

Surface characterization of the initial 500 nm Ni on Zr foil state (as-grown), observed by XPS, CO_2_ conversion profile on ultrapure Ni foil, CO desorption profiles from TPD analysis, TON and TOF calculation, time evolution of TON during reaction, CO_2_ conversion profiles of 1st, 2nd, 3rd, and 4th DRM cycle on 500 nm Ni–Zr, additional XPS profiles of the 100 nm Ni–Zr sample, maximum conversion percentages preregeneration, Ellingham diagram analysis, EDX spectra after the 4th DRM cycle, Ni particle size distribution after 4th DRM cycle post CO regeneration, and SEM image after 1st DRM cycle post-DRM (PDF)
