# Supplementary material for: Regeneration of Ni–Zr Methane Dry Reforming Catalysts in CO2: Reduction of Coking and Ni Redispersion
Source: ACS Catal. 2025 Feb 10;15(4):3314–27. doi: 10.1021/acscatal.4c06230 (PMC11851784; doi:10.1021/acscatal.4c06230)
Supplement: Supplementary file 1 — cs4c06230_si_001.pdf [file cs4c06230_si_001.pdf]

Supporting Information

Regeneration of Ni-Zr dry reforming catalysts in CO_2: reduction of coking and Ni re-dispersion

*Mahdi Hosseinpour, Toni Moser, Bernhard Klötzer, Simon Penner**

Institute of Physical Chemistry, University of Innsbruck, Innrain 52c, 6020 Innsbruck (Austria)

Corresponding author: Simon Penner*, simon.penner@uibk.ac.at, +4351250758003

Keywords: Catalyst Regeneration, Intermetallic Precursor, CO_2, Sintering, Carbon Deposition, Redispersion, Reverse Boudouard Reaction

Section A: Surface characterization of the initial 500 nm Ni on Zr foil state

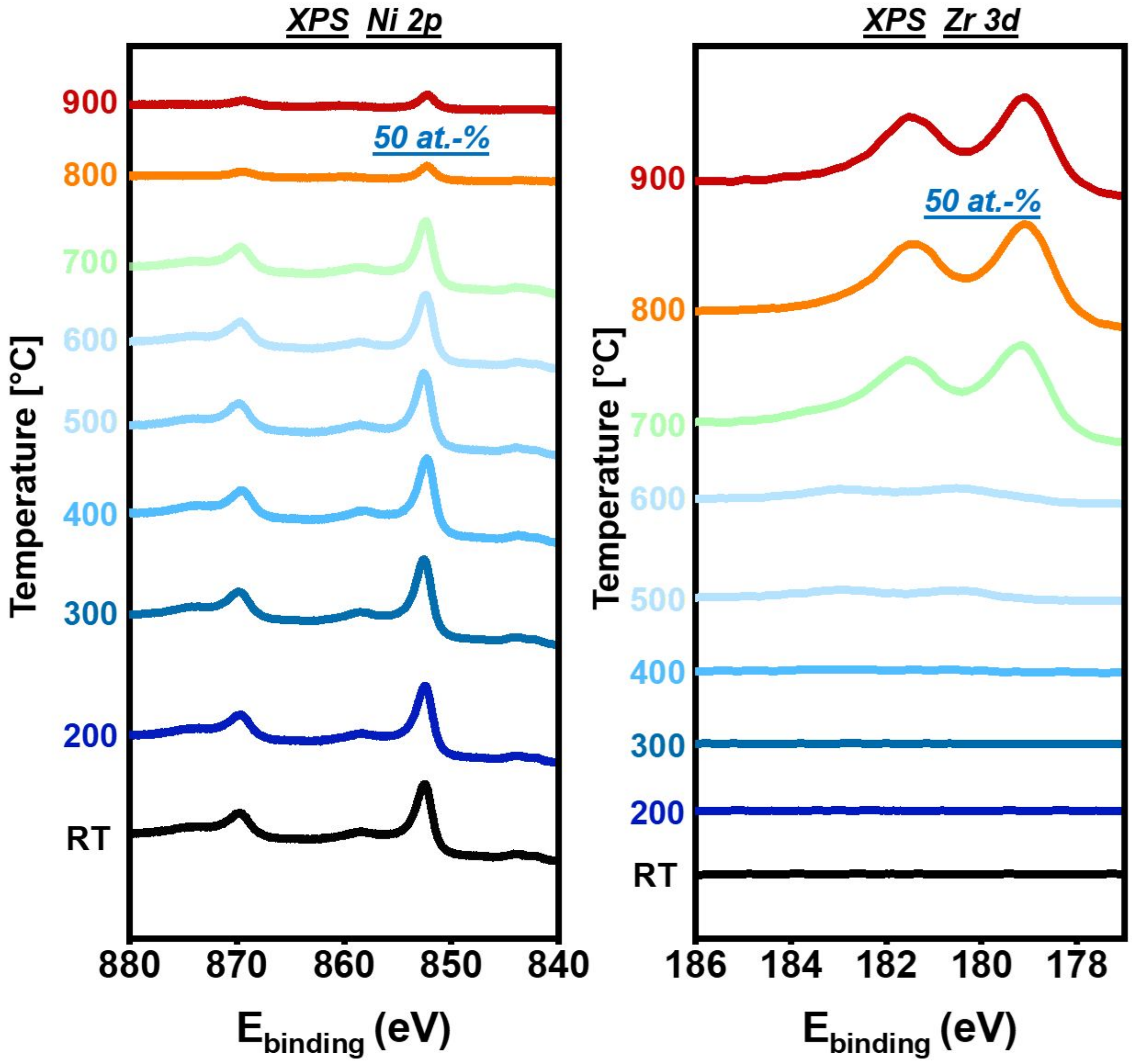

Figure S1. Surface characterization of the initial 500 nm Ni on Zr foil state (as-grown), observed by XPS during the annealing process as the temperature was increased from 25°C to 900°C.

Section B CO_2 conversion profile on ultra-pure Ni foil.

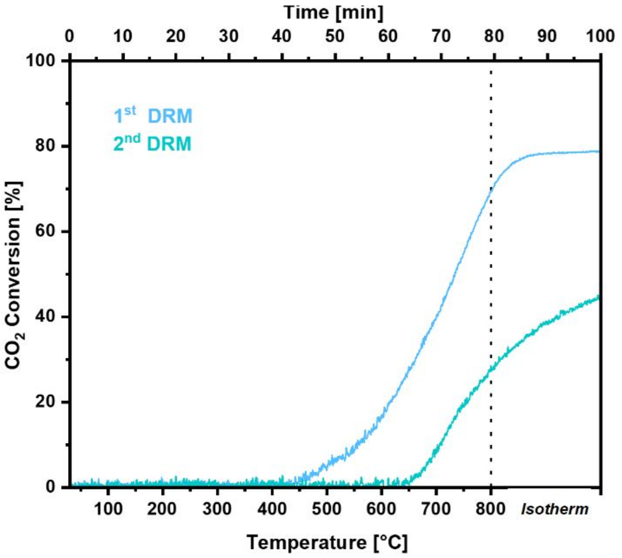

Figure S2. CO_2 conversion profile on ultra-pure Ni foil.

Figure S2 illustrates the CO_2 conversion profiles on initially ultra-pure Ni foil obtained during the 1st and 2nd DRM (dry reforming of methane) cycles. In the first cycle, a rapid increase in CO_2 conversion above 400°C is observed, reaching nearly 80%. During the second DRM cycle, the overall CO_2 conversion rate is noticeably lower, indicating a decrease in catalytic activity due to carbon deposition (coking). Moreover, the reaction onset temperature is shifted to 650°C due to the presence of Ni^0 surface site-blocking carbon deposits. This comparison highlights the deactivation of the ultra-pure Ni foil catalyst over only two DRM cycles in a row, emphasizing the need for an effective support or co-catalyst to increase the coking stability of the catalyst.

Section C Quantification of catalytic data from CO TPD analysis

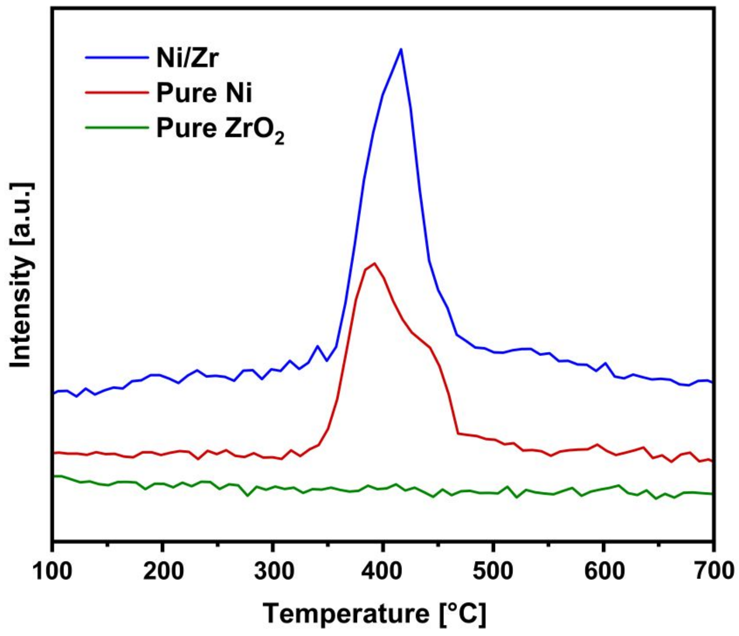

Figure S3. CO desorption profiles on the 100 nm Ni/Zr catalyst after CO_2 regeneration, on the pure Ni Foil, and the pure ZrO_2 foil.

The CO desorption profile of the Ni/Zr catalyst after CO_2 regeneration is shown in Figure S3. The TPD results emphasize the redistribution of Ni particles on the Zr support following CO_2 treatment. A prominent desorption peak indicates the availability of active Ni sites with enhanced CO adsorption capacity. The increased intensity of the TPD signal reflects a higher density of active sites, attributed to the redispersion of Ni particles facilitated by the CO_2 treatment. This redistribution minimizes sintering effects, leading to a more uniform and accessible catalytic surface and decreasing carbon deposition. These findings underline the importance of CO_2-induced regeneration in restoring and optimizing the catalyst structure for improved catalytic performance in DRM. The TPD data were used to quantify the number of active sites, which subsequently served as the basis for the turnover frequency (TOF) and turnover number (TON) calculations, detailed in the following.

Average Ni surface atom density of Ni foil, assumption of 1:1 ratio of (100) and (111) planes:

0.866 (atom density of (100) relative to (111)) $\rightarrow$ $\frac{0.866+1}{2} = 0.933$

$1.864 \times 10^{15} \times 0.933 = 1.739 \times 10^{15}$ Ni surface atoms cm^{-2}

1.739×10^{15} cm^{-2} $\times$ 7.2 cm^{2} = 1.252×10^{16} → total Number of surface atoms in the pure Ni sample foil

TPD peak area pure Ni foil = 1.553×10^{-9} a.u.

TPD peak area Ni/Zr catalyst = 2.625×10^{-9} a.u.

$$Number\ of\ active\ sites = \frac{1.252\times10^{16}\times2.625\times10^{-9}}{1.553\times10^{-9}} \sim 2.116 \times 10^{16}\ \text{cm}^{-2}$$

$$n_{initial} = \frac{P_{initial}\,.V_{reactor}}{\text{RT}}$$

$$n_{initial} = \frac{5000\ \text{Pa} \times 2.96 \times 10^{-4} m^3}{8.314\ \text{J.K}^{-1}\ \text{mol}^{-1} \times 298\ \text{K}} \sim 5.97 \times 10^{-4}\ mol\ CO_2$$

$$n_{Consumed} = n_{initial} \cdot X_{CO_2}$$

$$n_{Consumed} = 5.97 \times 10^{-4} \times 0.9 = 5.373 \times 10^{-4}\ mol$$

$$moles\ of\ CO_2\ \text{per sec} = \frac{n_{Consumed}}{\text{t}}$$

$$moles\ of\ CO_2\ \text{per sec} = \frac{5.373 \times 10^{-4}\ mol}{3000\ \text{sec}} = 1.791 \times 10^{-7}\ mol/s$$

$$Conversion\ of\ moles\ to\ molecules = 1.791 \times 10^{-7} \times 6.022 \times 10^{23} \sim 1.077 \times 10^{17}\ \text{sec}^{-1}$$

$$TOF = \frac{1.077 \times 10^{17}}{2.116 \times 10^{16}} \sim 5.08\ s^{-1} site^{-1}$$

$$molecules\ of\ CO_2\ consumed = 5.373 \times 10^{-4} \times 6.022 \times 10^{23} \sim 3.23 \times 10^{20}$$

$$TON = \frac{3.23 \times 10^{20}}{2.116 \times 10^{16}} \sim 1.5 \times 10^{4}\ site^{-1}$$

Table 1. Time evolution of turnover number (TON) and its first derivative during the reaction.

Time (s)	*TON*	*1st derivative of TON (s^{-1})*
**360**	1.7×10^3	4.5
**660**	3×10^3	6.3
**900**	5×10^3	8.3
**1020**	6×10^3	9.7
**1380**	1×10^4	11.1
**1560**	1.2×10^4	7.6
**1800**	1.3×10^4	2.9
**3000**	1.5×10^4	1.6

The first derivative of TON, representing the instantaneous turnover frequency (TOF), reaches a maximum value of 11.1 s^{-1} at 1380 s, indicating the highest catalytic activity during the reaction cycle.

Section D CO_2 conversion profiles of 1st,2nd, 3rd, and 4th DRM cycles on the Ni/Zr catalyst with 500 nm deposition thickness

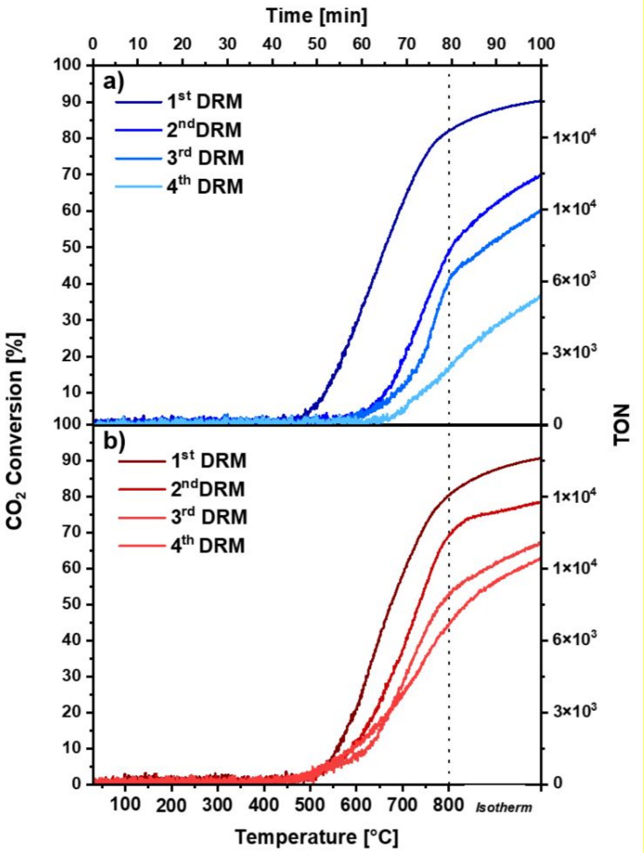

Figure S4. CO_2 conversion profiles of 1st,2nd, 3rd, and 4th DRM cycles on the Ni/Zr catalyst with 500 nm deposition thickness; a) before CO_2 regeneration; b) after CO_2 regeneration.

Figure S4 presents the CO_2 conversion profiles on the Ni/Zr catalyst with a 500 nm deposition thickness for four subsequent DRM cycles, comparing the catalyst's performance before and after CO_2 titration and highlighting the analogous regeneration effectiveness. While the initial CO_2 conversion declines with multiple DRM cycles due to pronounced deactivation, CO_2 regeneration 1) effectively restores the catalyst's initial activity and 2) obviously enhances the operational longevity and long-term coking stability of the catalyst, indicating a potentially useful application for practical DRM reactor operation.

Section E Additional XPS analysis on the 100 nm Ni-Zr sample after selected treatments

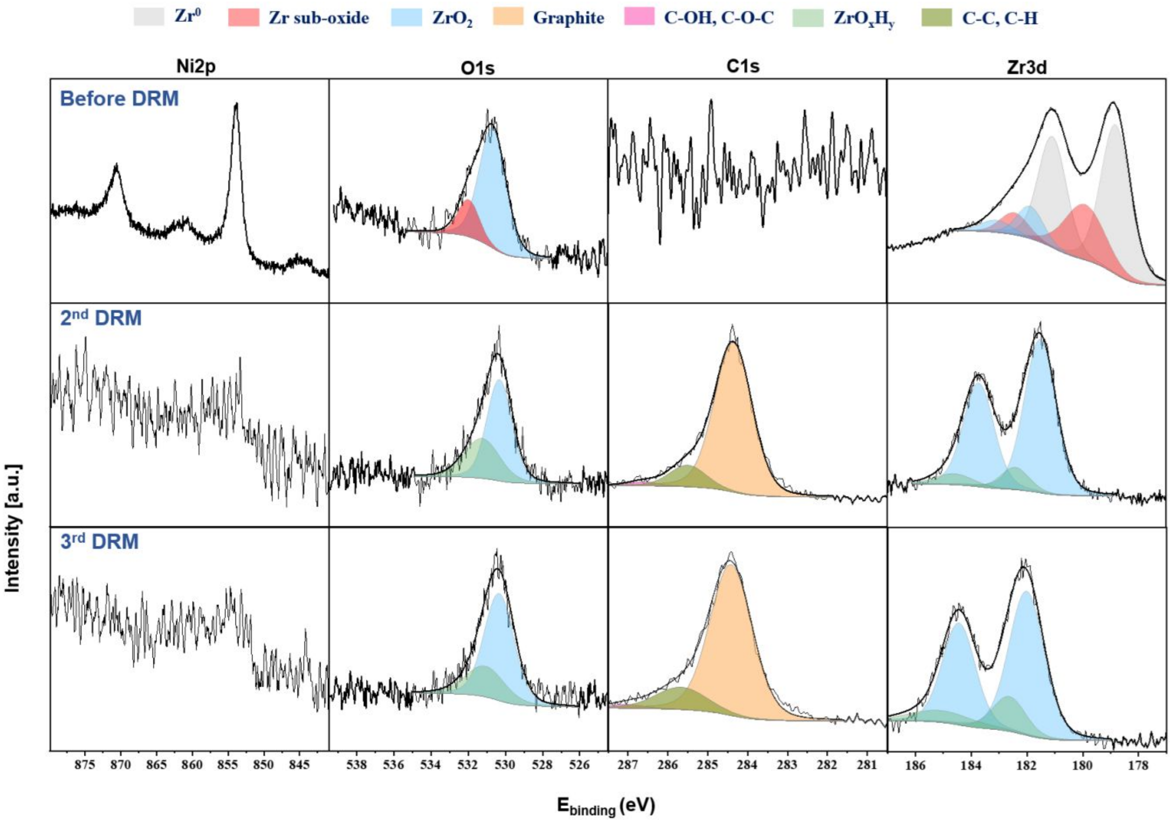

Figure S5. Ni2p, O1s, C1s, and Zr3d XPS spectra of the thermally annealed 100 nm Ni/Zr sample before DRM and after the 2nd and 3rd DRM cycles, obtained under UHV conditions.

Figure S5 displays the Ni2p, O1s, C1s, and Zr3d XPS spectra of the 100 nm Ni/Zr sample obtained under UHV conditions after thermal annealing and two subsequent DRM cycles. In the state before DRM, both Zr 3d and O 1s show contributions of ZrO_2 and Zr sub-oxides. The latter component is removed by DRM operation. Comparing these spectra with Figures 3i and 3j in the manuscript reveals hardly any differences. Before DRM there is a sharp peak of Ni and no carbon contamination, immediately after the first DRM cycle, a large amount of carbon appears on the catalyst surface, and the amount of nickel decreases. This suggests that the active sites of the catalyst surface are being enclosed by carbon after the first cycle of DRM, preventing the appearance of nickel on the surface. This carbon encapsulation finally

leads to catalyst deactivation after four cycles. These changes are also observed across all DRM cycles, emphasizing the impact of carbon deposition on catalyst performance.

Section F CO_2-conversion percentages pre-regeneration

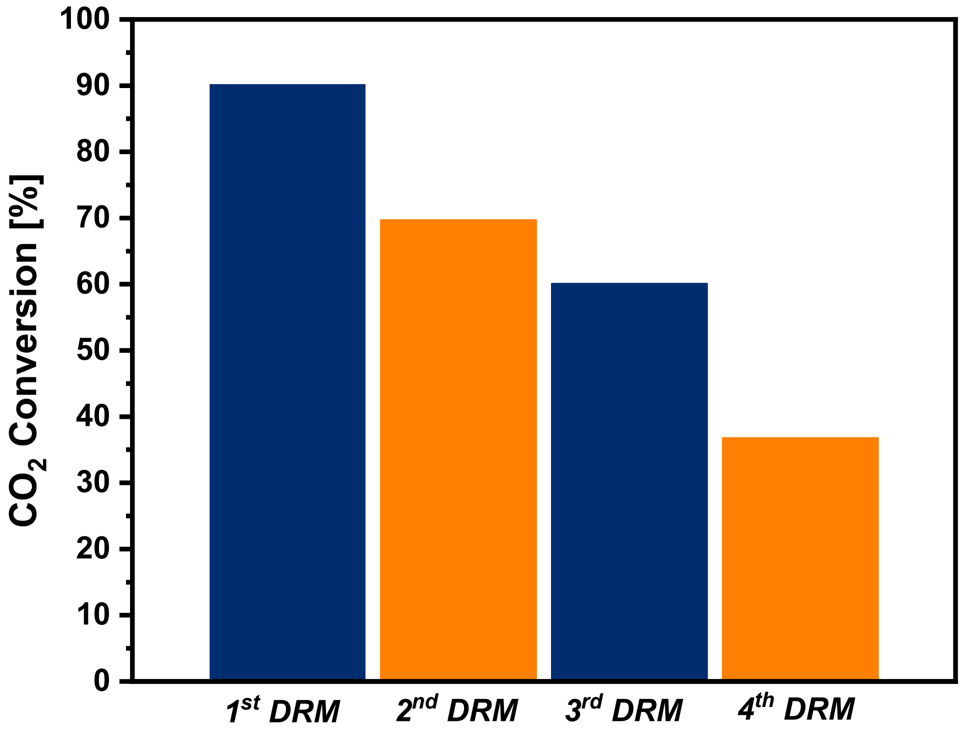

Figure S6. Maximum CO_2 conversion percentages (pre-regeneration) for the Ni/Zr catalyst across four DRM cycles in a row.

The bar chart in Figure S6 illustrates the gradual decrease in CO_2 conversion from the 1st DRM cycle to the 4th DRM cycle, emphasizing the deactivation trend of the catalyst over multiple reactions. The decreasing conversion efficiency underscores the impact of coke deposition on the catalyst's activity.

Section G Ellingham diagram analysis

The Ellingham diagram[1] can be used to calculate the desired value of ΔG as shown below.

$$\Delta G° = \ -RT\ LnKp$$

$\Delta G° \approx \ 40\frac{KJ}{mol}\ or\ 40000\frac{J}{mol}$

$R = \ 8.314\ \frac{J}{mol}$

$T = \ 700°\text{C}\ or\ 973K$

$$40000 = -(8.314\ \times 973)\ LnKp$$

$$40000 = -8093.522\ LnKp$$

$$Ln\ Kp = \frac{40000}{-8093.522} = \ -4.943$$

$$\text{Kp} = e^{-4.943} \approx 0.071$$

$$Kp = \frac{P^2CO}{\text{P}CO_2} \rightarrow 0.0071 = \ \frac{P^2CO}{PCO_2}$$

$$\left(\frac{PCO}{PCO_2}\right)^2 = 0.0071 \rightarrow \ \frac{\text{P}CO}{\text{P}CO_2} = \ \sqrt{0.0071}$$

$$\frac{PCO}{\text{P}CO_2} \approx 0.084$$

The final CO_2 ratio is approximately 0.084:1, which means that for each part of CO_2, there are about 0.084 parts of CO. This corresponds to 8.4% CO and 91.6% CO_2.

Section H EDX spectra of bimetallic Ni-Zr after the 4th post CO_2 regeneration DRM cycle

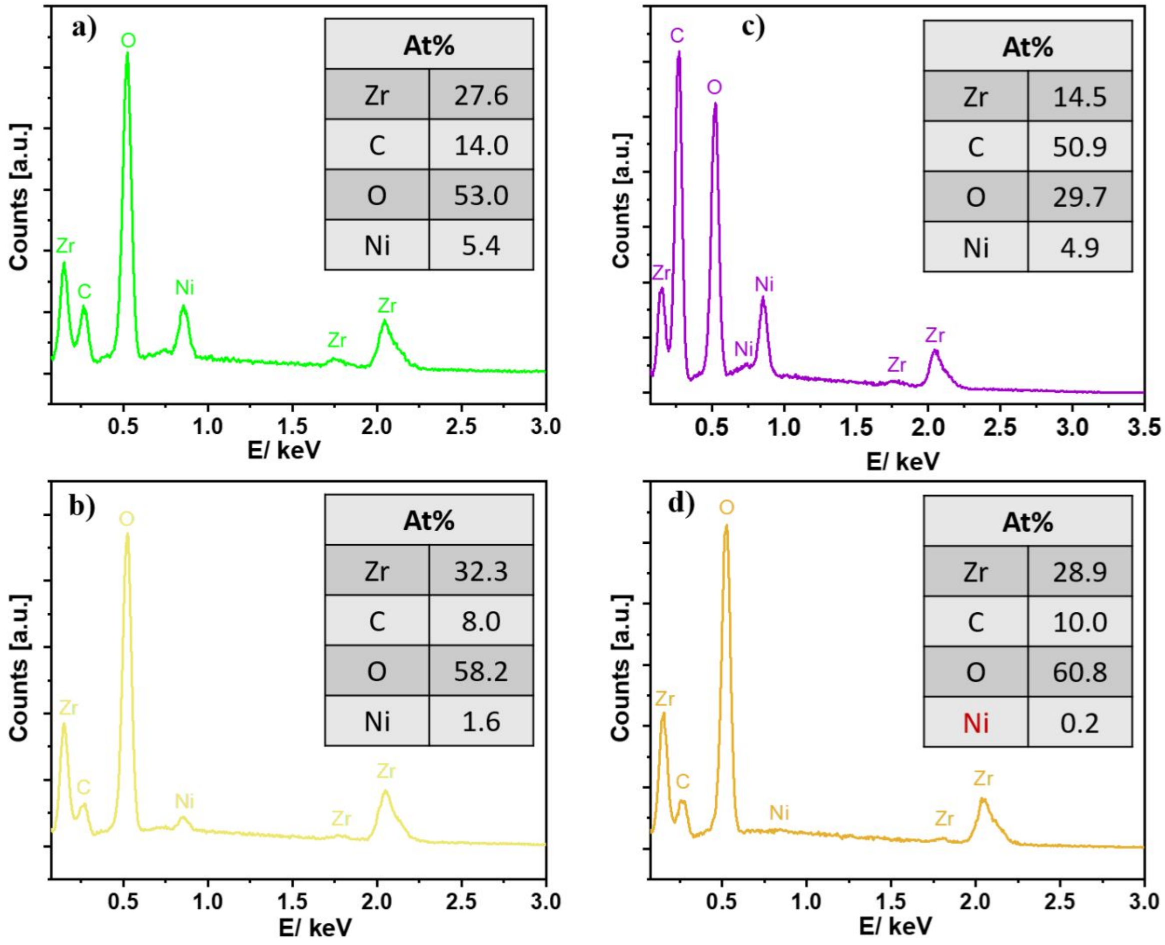

Figure S7. EDX spectra of bimetallic Ni-Zr after the 4th post CO_2 regeneration DRM cycle, highlighting the elemental composition in different "breakout" regions. Assignment: a) green, b) yellow, c) purple, d) orange-marked regions of Figure 7c.

Section I Ni particle size distribution after the 4th DRM cycle post-CO_2 treatment

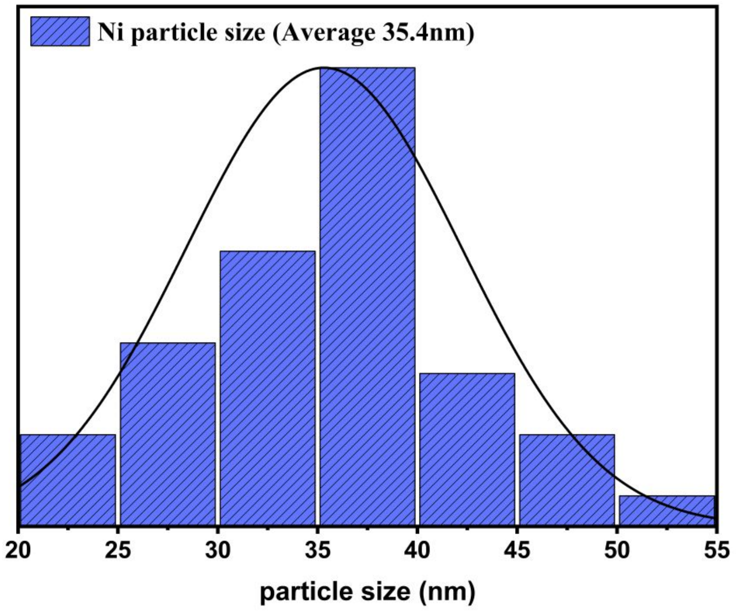

Figure S8. Ni particle size distribution after the 4th DRM cycle post-CO_2 treatment.

According to Figure S8, the results reveal a uniform particle size distribution with an average size of ~35 nm, demonstrating the effectiveness of the CO_2 treatment in redistributing Ni particles and mitigating sintering effects. The absence of a bimodal size distribution, which was observed before the CO_2 treatment (Figure 5 main manuscript), further supports the conclusion that CO_2 treatment plays a critical role in breaking down larger aggregates and redistributing Ni particles into a more uniform size. This uniformity enhances catalytic performance by increasing the fraction of smaller, highly active particles while simultaneously reducing the number of inactive or encapsulated larger particles. Larger Ni particles are still observed, but as shown in the SEM images are substantially covered by ZrO_2 (cf. Figure 8) and/or act as source for the fragmentation in smaller Ni domains.

Section J Structural characterization of the 100 nm Ni-Zr sample after the 1st post-CO_2 treatment DRM cycle

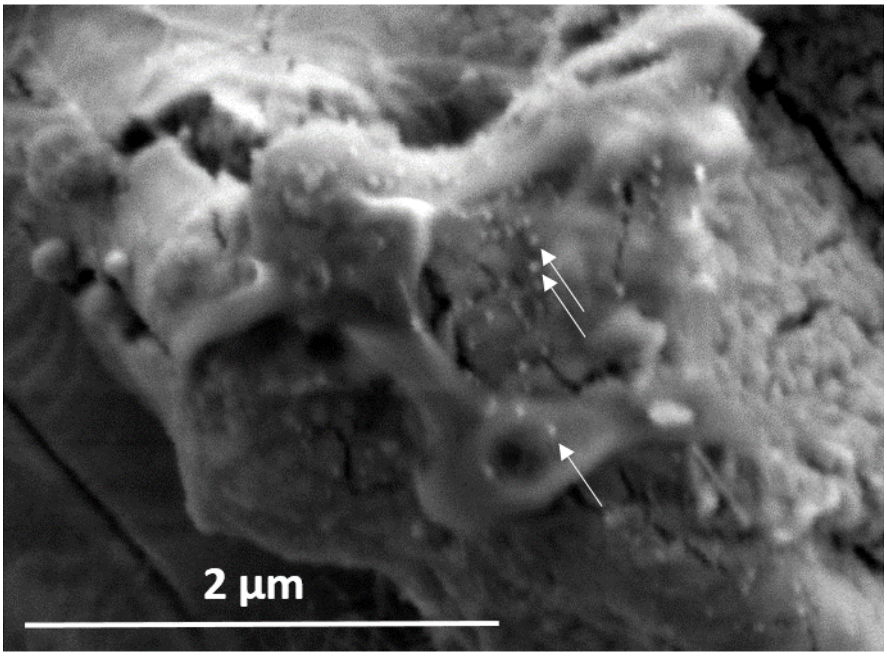

Figure S9: SEM image on the 100 nm Ni-Zr sample after the 1st post-CO_2 treatment DRM cycle. Three Ni particles are highlighted by arrows.

References

(1) Yu, H.; Hayashi, S.; Kakehi, K.; Kuo, Y.-L. Study of formed oxides in IN718 alloy during the fabrication by selective laser melting and electron beam melting. *Metals* **2018**, *9*, 19.
